# Role of transient receptor potential ankyrin 1 in idiopathic pulmonary fibrosis: modulation of M2 macrophage polarization

**DOI:** 10.1007/s00018-024-05219-x

**Published:** 2024-04-18

**Authors:** Yi Yang, Zhenyu Xiao, Weijie Yang, Yangyang Sun, Xin Sui, Xueyang Lin, Xinyi Yang, Zhenghao Bao, Ziqi Cui, Yingkai Ma, Weidong Li, Shengran Wang, Jun Yang, Yongan Wang, Yuan Luo

**Affiliations:** State Key Laboratory of Toxicology and Medical Countermeasures, Beijing Institute of Pharmacology and Toxicology, Beijing, China

**Keywords:** Idiopathic pulmonary fibrosis, Transient receptor potential ankyrin 1, Macrophages, Polarization, Transforming growth factor-β1

## Abstract

Idiopathic pulmonary fibrosis (IPF) poses significant challenges due to limited treatment options despite its complex pathogenesis involving cellular and molecular mechanisms. This study investigated the role of transient receptor potential ankyrin 1 (TRPA1) channels in regulating M2 macrophage polarization in IPF progression, potentially offering novel therapeutic targets. Using a bleomycin-induced pulmonary fibrosis model in C57BL/6J mice, we assessed the therapeutic potential of the TRPA1 inhibitor HC-030031. TRPA1 upregulation was observed in fibrotic lungs, correlating with worsened lung function and reduced survival. TRPA1 inhibition mitigated fibrosis severity, evidenced by decreased collagen deposition and restored lung tissue stiffness. Furthermore, TRPA1 blockade reversed aberrant M2 macrophage polarization induced by bleomycin, associated with reduced Smad2 phosphorylation in the TGF-β1-Smad2 pathway. In vitro studies with THP-1 cells treated with bleomycin and HC-030031 corroborated these findings, highlighting TRPA1's involvement in fibrotic modulation and macrophage polarization control. Overall, targeting TRPA1 channels presents promising therapeutic potential in managing pulmonary fibrosis by reducing pro-fibrotic marker expression, inhibiting M2 macrophage polarization, and diminishing collagen deposition. This study sheds light on a novel avenue for therapeutic intervention in IPF, addressing a critical need in the management of this challenging disease.

**Graphical abstract:**

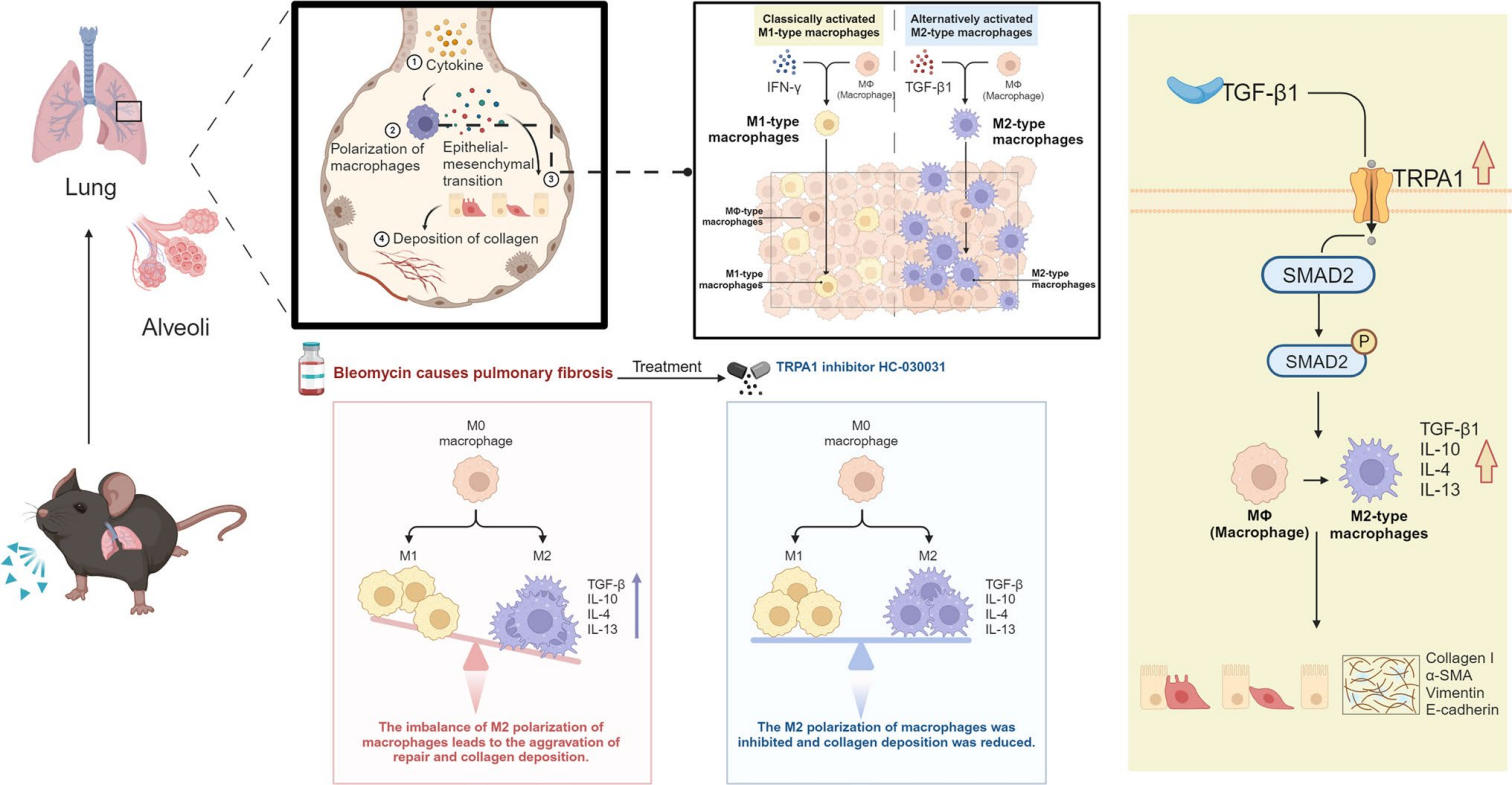

**Supplementary Information:**

The online version contains supplementary material available at 10.1007/s00018-024-05219-x.

## Introduction

Idiopathic pulmonary fibrosis (IPF) is a challenging and enigmatic disorder characterized by progressive fibrosis of the lung parenchyma that ultimately leads to respiratory failure [1]. Despite ongoing research, the exact cause of IPF remains unknown, which poses a significant global health threat. The development of IPF is the result of a multifaceted interplay that includes genetic predisposition, environmental factors, and aberrant wound-healing responses. Common symptoms include shortness of breath, chronic cough, and fatigue. The prevalence of IPF has been steadily increasing worldwide, requiring global efforts to comprehensively understand and treat this incapacitating ailment [2, 3]. There are two approved drugs, nintedanib and pirfenidone, which offer potential for managing of this challenging condition [4, 5]. Unfortunately, a cure for IPF is not yet known, current treatments mainly focus on symptom management and slowing disease progression [6]. Thus, our research aimed to investigate the underlying mechanisms of this disorder.

Macrophages play a significant role in the regulation of pulmonary fibrosis by differentiating into distinct functional phenotypes. M1-polarized macrophages, known for their pro-inflammatory activity, contribute to the initial stages of fibrosis by releasing inflammatory cytokines [7]. The M2 phenotype of macrophages, primarily involved in tissue remodeling and repair, play a crucial role in IPF [8, 9]. These M2 macrophages facilitate collagen deposition by secreting anti-inflammatory cytokines and growth factors, contributing significantly to the fibrotic process. The transforming growth factor (TGF)-β1-mediated suppressor of mothers against decapentaplegic 2 (Smad2) signaling pathway, which has been shown to modulate macrophage polarization, is critical in this process [10, 11]. This pathway is recognized for its pro-fibrotic activities such as regulating myofibroblast differentiation and extracellular matrix production.

Transient receptor potential (TRP) channels are integral membrane proteins that act as ion channels and play a crucial role in various physiological processes [12–14]. One particular TRP channel, known as transient receptor potential ankyrin 1 (TRPA1), can be activated by various physical or chemical stimuli[15, 16]. Activation of TRPA1 leads to the influx of calcium ions and subsequently triggers signaling pathways that can potentially modify the function and phenotype of macrophages [17]. In a study on scleroderma dermal fibrosis, markers of M2-type macrophage activation, which are crucial for tissue fibrosis, were reduced in TRPA1-deficient mice treated with bleomycin [18]. A study on cardiac hypertrophy and fibrosis discovered that TRPA1 inhibition protected against cardiac hypertrophy and suppressed cardiac dysfunction through Ca^2+^-dependent signaling pathways and inhibition of M2 macrophage transition [19]. Additionally, TRPA1 was found to be involved in TGF-β1 signaling, and the absence of TRPA1 in cultured ocular fibroblasts decreased the expression of TGF-β1 and related pro-inflammatory and pro-fibrotic markers [20].

In summary, the regulation of TRPA1 channels by TGF-β1 suggests that TRPA1 may play a key role in macrophage polarization, which can affect the balance between the M1 and M2 phenotypes and influence the progression of pulmonary fibrosis. Understanding the intricate mechanisms underlying macrophage polarization and the role of TRPA1 channels are important for developing novel therapeutic strategies for IPF. This study demonstrated that inhibiting TRPA1 channels can decrease pulmonary fibrosis in mice. TRPA1 has an impact on M2-type macrophage polarization and the activation of the TGF-β1-Smad2 pathway, which is crucial for fibrosis development. Therefore, TRPA1 inhibition may provide a novel therapeutic strategy for IPF by influencing macrophage dynamics and fibrosis-related pathways.

## Materials and methods

### Reagents

Bleomycin (Cat# HY-17565, ≥ 98.53% purity) and TRPA1 antagonist HC-030031 (Cat# HY-15064,  ≥ 95.91% purity) were purchased from MedChemExpress (Monmouth Junction, NJ, USA).

### Animals

Experiments involving animals were conducted in accordance with national legislation and approved by the Institutional Animal Care and Use Committee (IACUC number: IACUC-DWZX- 2023-579, Laboratory Animal Center of the Academy of Military Medical Science, Beijing, China).

Male C57BL/6J mice, aged 8–10 weeks and weighing 20.5–23.5 g, were acquired from SPF Biotechnoloy Co., Ltd., Beijing, China. The animals were maintained in a controlled environment with regulated temperature and humidity. The mice were housed in a room set at 23 ± 2 ℃ with a 12-h light/dark cycle and had ad libitum access to food and water.

### Cell culture

THP-1 cells were obtained from the Peking Union Medical College Hospital Cell Bank and cultured in Roswell Park Memorial Institute 1640 medium supplemented with 10% heat-inactivated fetal bovine serum (Gibco, Carlsbad, CA, United States) at 37 °C in a 5% CO_2_ atmosphere. For experiments involving whole protein or mRNA extraction and subsequent fluorescence analysis, cells were seeded in 6-well or 24-well glass plates (Cellvis, Mountain View, CA, USA). The THP-1 cells were divided into four groups: Control group, cultured in standard complete medium; HC-030031 control group, cultured with medium containing 10 μM HC-030031; Bleomycin group, cultured with medium containing 10 μg/mL bleomycin; and Bleomycin + HC-030031 group, cultured with medium containing both 10 μg/mL bleomycin and 10 μM HC-030031 (n = 3 for each group).

### IPF mouse model and treatment

The animals were divided into saline control group, HC-030031 control group, bleomycin-treated group and bleomycin +HC-030031 treated- group. We allocated a portion of the animals in each group for behavioral assessments and histopathological examinations, while others were designated for the collection of samples for molecular analysis.

Bleomycin-treated mice received aerosolized bleomycin (3 mg/kg) on the first day, and the bleomycin + HC-030031 group received an intraperitoneal injection of HC-030031 (10 mg/kg) one hour earlier and were intraperitoneally injected once a week during the modeling period [21, 22]. Throughout the experiment, the mouse body weights were recorded daily, and their survival was monitored. Lung function was assessed every seven days using Whole-Body Plethysmography (EMKA Technologies, Paris, France). On the 21st day, lung computed tomography (CT) scans were performed using a Quantum GX_2_ small-animal in vivo imaging system (PerkinElmer, Waltham, MA, USA), and arterial blood gas indices were measured using an ABL800 FLEX blood gas analyzer (Radiometer, København, Denmark). At the endpoint, the mice were euthanized, and their lung tissues were collected for histological examination, collagen quantification, and molecular analyses (n = 6 per group).

### Hematoxylin and eosin (HE) staining of lung tissue sections

The lung tissue sections were subjected to HE staining to reveal the histological details. The tissues were fixed in 4% neutral polymerization formaldehyde for 48 h, embedded in paraffin, and 4-μm sections were made using a microtome. Deparaffinization was achieved using xylene, followed by rehydration in descending ethanol concentrations and rinsing with distilled water. Hematoxylin staining was performed for 3 min using acid alcohol differentiation and eosin counterstaining. Dehydration in ascending ethanol concentrations and clearing in xylene were performed to complete the staining process. Microscopic examination of HE-stained sections involved evaluating cellular architecture, identifying inflammatory infiltrates, and assessing overall tissue integrity. Digital images capturing representative fields were acquired using a Nikon Eclipse Ci (Tokyo, Japan), and a 3DHistech Case Viewer (Budapest, Hungary) was used for panoramic tissue scanning. The methodology adhered to standardized protocols to ensure consistency. This systematic approach provided comprehensive insights into pulmonary histopathology through HE staining, forming the basis for subsequent analyses (n = 6 per group).

### Masson's trichrome staining of lung tissue sections

Lung tissue sections were subjected to Masson's trichrome staining to assess the collagen distribution and fibrotic changes. The staining protocol involved immersion in Weigert's iron hematoxylin and differentiation in Biebrich scarlet acid fuchsin. Aniline blue staining was performed, and the sections were differentiated in a phosphomolybdic–phosphotungstic acid solution. Subsequently, the sections were immersed in an aniline blue solution, followed by acetic acid treatment. The staining process was completed by dehydration in ethanol, clearing in xylene, and mounting. Microscopic examination focused on collagen fibers, highlighting connective tissue and fibrotic areas. Digital images of representative fields were captured using a Nikon Eclipse Ci, and 3DHistech Case Viewer was used for panoramic tissue scanning. Blue collagen in Masson staining was quantified using the HALO Digital pathology image Analysis tool (lndica Labs, USA, n = 6 per group).

### Sirius red staining of lung tissue sections

This study used Sirius Red staining and polarized light microscopy to observe and analyze lung tissue samples. The staining process involved immersing the samples in Sirius Red for a specified duration, followed by differentiation in acidified water. Counterstaining was performed with hematoxylin and the sections were dehydrated and cleared in xylene. Microscopic examination focused on the collagen fibers enhanced by Sirius Red staining. Digital images of representative fields were acquired using a Nikon Eclipse Ci microscope, and panoramic tissue scans were performed using a 3DHistech Case Viewer. Red collagen in Sirius Red staining was quantified using the HALO Digital pathology image Analysis tool (lndica Labs, USA). The stained sections were observed under a polarized light microscope. This method enabled specific visualization of collagen fibers, which appeared bright red and were distinctly highlighted against a pale-yellow background. The birefringence of collagen facilitated the use of polarized light to analyze the architecture and collagen distribution (n = 6 per group).

### Atomic force microscope detection

We utilized atomic force microscopy (AFM) to explore the nanoscale morphology and mechanical attributes of biological specimens. Tissues were thinly sectioned and affixed to mica substrates with biocompatible glue, followed by air drying prior to analysis. AFM scanning was executed in tapping mode using a silicon nitride probe (spring constant: 0.01 N/m) to preserve sample integrity, with parameters optimized for high-resolution imaging and minimal tip-sample interaction. Analysis of AFM outputs provided insights into surface roughness, particle dimensions, and other structural characteristics. Mechanical properties, including stiffness and elasticity, were evaluated through force-distance curve analysis, with the effective Young's modulus gauging cellular rigidity linked to cytoskeletal and membrane firmness. Force spectrum analysis was performed at 10 points in three randomly selected tissue sections to compare the elastic modulus of lung tissue in each group.

### Enzyme-linked immunosorbent assay (ELISA)

ELISA kits (Meimian, Jiangsu Meimian Industrial Co., Ltd., Jiangsu, China) were used to detect serum interleukin (IL)-4, IL-10, and IL-13 protein levels in mice (n = 6 per group). Standard and serum samples were added to pre-coated samples with traces of specific antibodies. After incubation and washing to remove the unbound material, biotinylated antibodies against the target proteins were added. After incubation and washing, the streptavidin–horseradish peroxidase conjugate was added. The reaction used the 3,3′,5,5′-tetramethylbenzidine substrate and was stopped with sulfuric acid. The absorbance was measured at 450 nm using a microplate reader (RT-6100, Rayto Life and Analytical Sciences Co., Ltd., Shenzhen, China). Data were analyzed using a standard curve for quantitative protein concentration.

### Flow cytometry analysis

Lung tissues were harvested, minced, and enzymatically digested in collagenase solution (BS165, Biosharp, Hefei city, China) at 37 °C for 30 min. The tissues were processed through a 70-μm cell strainer post-digestion to achieve a single-cell suspension. The resulting cells were washed and resuspended in phosphate-buffered saline. Alveolar macrophages were differentiated using Brilliant Violet™ (BV)421 anti-mouse F4/80 (565411; BioLegend, San Diego, CA, USA), BV605 anti-mouse cluster of differentiation (CD)11b; 101257; BioLegend), and fluorescein isothiocyanate (FITC) anti-mouse major histocompatibility complex II (107605; BioLegend). M1 and M2 macrophages were differentiated using allophycocyanin anti-mouse CD86 (105012, BioLegend) and phycoerythrin anti-mouse CD206 (141706, BioLegend) antibodies, respectively. Cells were incubated with fluorescently conjugated antibodies targeting specific cell surface markers for 30 min at 4 °C in darkness for immunofluorescent staining. After staining, the cells were washed, fixed, and subjected to flow cytometric analysis using a BD LSRFortessa™ flow cytometer (Becton, Dickinson and Company, Franklin, NJ, USA) (n = 3–5 mice per group).

### Real-time quantitative polymerase chain reaction (RT-qPCR)

Total RNA was extracted from the lung tissue or cells using TRIzol reagent (Invitrogen, Carlsbad, CA, USA). Using PrimeScript ™ RT Reagent Kit with genomic DNA Eraser reverse transcription (Takara, Osaka, Japan) in accordance with the specification. TB Green®Premix Ex Taq™II (Takara, Osaka, Japan) and Bio-Rad CFX96 real-time polymerase chain reaction (PCR) assay System (BioRad Laboratories Ltd., Hercules, CA, USA) were used for real-time quantitative PCR analysis. Amplification was performed as follows: 40 cycles of 95 °C for 30 s, 95 °C for 15 s, 60 °C for 30 s, and 72 °C for 30 s. Primer sequences for the target genes are listed in Table 1.

**Table 1:** Oligo nucleotide sequences of the primers used for quantitative (qPCR)

Gene	Forward (5' to 3')	Reverse (5' to 3')
TRPA1	AGTATATTTGGGTATTGCAAAGAAGC	ATGCCCGTCGTGTAGATAATCC
CD206	TCCGGGTGCTGTTCTCCTA	CCAGTCTGTTTTTGATGGCACT
CD163	TTTGTCAACTTGAGTCCCTTCAC	TCCCGCTACACTTGTTTTCAC
GAPDH	AACGGATTTGGTCGTATTG	GCTCCTGGAAGATGGTGAT

*TRPA1* transient receptor potential ankyrin 1, *CD* cluster of differentiation, *GAPDH* glyceraldehyde 3-phosphate dehydrogenase

### Western blot

To extract proteins, mouse lung tissues or cultured cells were homogenized using lysis buffer (KeyGEN, Nanjing, China) containing protease and phosphatase inhibitors. The mixture was then lysed on ice for 30 min, and centrifuged at 12,000 rpm for 10 min at 4 °C. The supernatant was collected and quantified using a Bicinchoninic Acid Protein Quantification kit (KeyGEN, Nanjing, China), mixed with 5 × sodium dodecyl-sulfate (SDS) loading buffer, and heated at 100 °C for 15 min. Whole proteins were extracted and separated using 10% SDS-polyacrylamide gel electrophoresis, and then transferred to a 0.22-μm polyvinylidene fluoride membrane. After blocking with 5% bovine serum albumin (BSA) for 2 h at room temperature (RT), the membranes were incubated with primary antibodies overnight at 4 °C, washed with Tris-buffered saline with 0.1% Tween® 20 Detergent, and incubated with appropriate horseradish peroxidase-coupled secondary antibodies (TianGen Biotech, Beijing, China) for 2 h at RT. The primary antibodies used in this study were anti-β-actin (1:1000, ab8226, Abcam, Cambridge, UK), anti-α-smooth muscle actin (SMA) (1:1000, ab7817, Abcam), anti-vimentin (1:1000, ab92547, Abcam), anti-E-cadherin (1:1000, ab40772, Abcam), anti-collagen I(1:1000, ab34710, Abcam), anti-Smad2 (1:1000, ab33875, Abcam), anti-phospho-Smad2 (1:1000, ab216482, Abcam), anti-TGF-β1 (1:1000, ab215715, Abcam), anti-TRPA1 (1:1000, PA1-46159, Thermo Fisher Scientific Inc., Waltham, MA, USA), anti-IL-4 (1:1000, AF5142, Affinity Biosciences, Cincinnati, OH, USA), anti-IL-13 (1:1000, DF6813, Affinity Biosciences), and anti-CD206 (1:1000, DF4149, Affinity Biosciences). Protein bands were visualized using a Bio-Rad GelDoc XR^+^ chemiluminescence system, and optical density analysis was performed using Image Lab software (Bio-Rad).

### Immunohistochemical staining

The tissue sections were deparaffinized in a gradient, dehydrated in ethanol, and microwaxed in citrate buffer (Beijing Zhongshan Jinqiao Biological Technology Co., Ltd., Beijing, China) for 30 min for antigen repair. The sections were then cooled to RT and incubated in 3% hydrogen peroxide to quench the endogenous peroxidase activity. Nonspecific binding was blocked with 5% BSA for 1 h. Primary antibodies were applied overnight at 4 °C using: anti-α-SMA (1:200), anti-vimentin (1:200), anti-E-cadherin (1:200), anti-CD206 (1:300), anti-IL-10 (1:200), anti-IL-4 (1:200), and anti-IL-13 (1:200). After washing, biotinylated secondary antibodies were added for 1 h at RT. Visualization was achieved using 3,3′-diamino-benzidine substrate, and sections were counterstained with hematoxylin. Digital images with representative fields were obtained for tissue sections using a Nikon Eclipse Ci, and the cells were photographed using an Image Xpress Micro Confocal system (United States). The immune signal intensity was quantified semi-automatic using Image-Pro Plus software, and random six images were counted for each mouse, as described previously.

### Immunofluorescence staining

After dewaxing and dehydration, the mouse lung tissue sections were permeabilized with 0.3% Triton X-100 and blocked with 5% BSA for 1 h at RT. Sections were incubated overnight with specific primary antibodies: anti-E-cadherin (1:200), anti-TRPA1 (1:200), anti-fibronectin (1:200, ab2413, Abcam), anti-CD206 (1:200), and anti-CD86 (1:200). The slides were then treated with appropriate secondary antibodies: Alex Fluor™647 goat anti-mouse immunoglobulin (Ig)G heavy chain + light chain (H + L) (1:1000, ab150115, Abcam); Alex Fluor™488 goat anti-rabbit IgG (H + L) (1:1000, ab150077, Abcam); and Alex Fluor™594 goat anti-rat IgG (H + L) (1:1000, ab150167, Abcam) for 1 h. After incubation with 5 μg 4′,6-diamidino-2-phenylindole, tissue sections were washed, mounted, and dried before imaging. The cells were fixed with 4% paraformaldehyde for immunofluorescence detection using the procedure described above. Digital images with representative fields were obtained for tissue sections using a Nikon Eclipse Ci, and the cells were photographed using an Image Xpress Micro Confocal system. The immune signal intensity was quantified semi-automatically using Image-Pro Plus software, and six random images were counted for each mouse, as described previously.

### Statistical analysis

Results are expressed as means ± standard deviation (SD). The Student's t-test was used for statistical analysis. One-way analysis of variance and Tukey's post hoc test were used for comparisons among multiple groups, and *P* < 0.05 was considered as statistically significant.

## Results

### Inhibition of TRPA1 attenuated bleomycin-induced lung dysfunction in mice.

To elucidate TRPA1’s involvement in IPF, we analyzed its expression in lung tissues from mice subjected to bleomycin-induced fibrosis. Western blot analysis revealed a significant upregulation of TRPA1 protein levels in bleomycin-exposed mice compared with that in controls (Figure S1A). Consistently, RT-qPCR demonstrated a marked increase in TRPA1 mRNA levels in the lungs of bleomycin-treated mice (Figure S1B). These results indicate a potential contributory role of TRPA1 in the pathogenesis of bleomycin-induced pulmonary fibrosis.

**Fig. 1:**
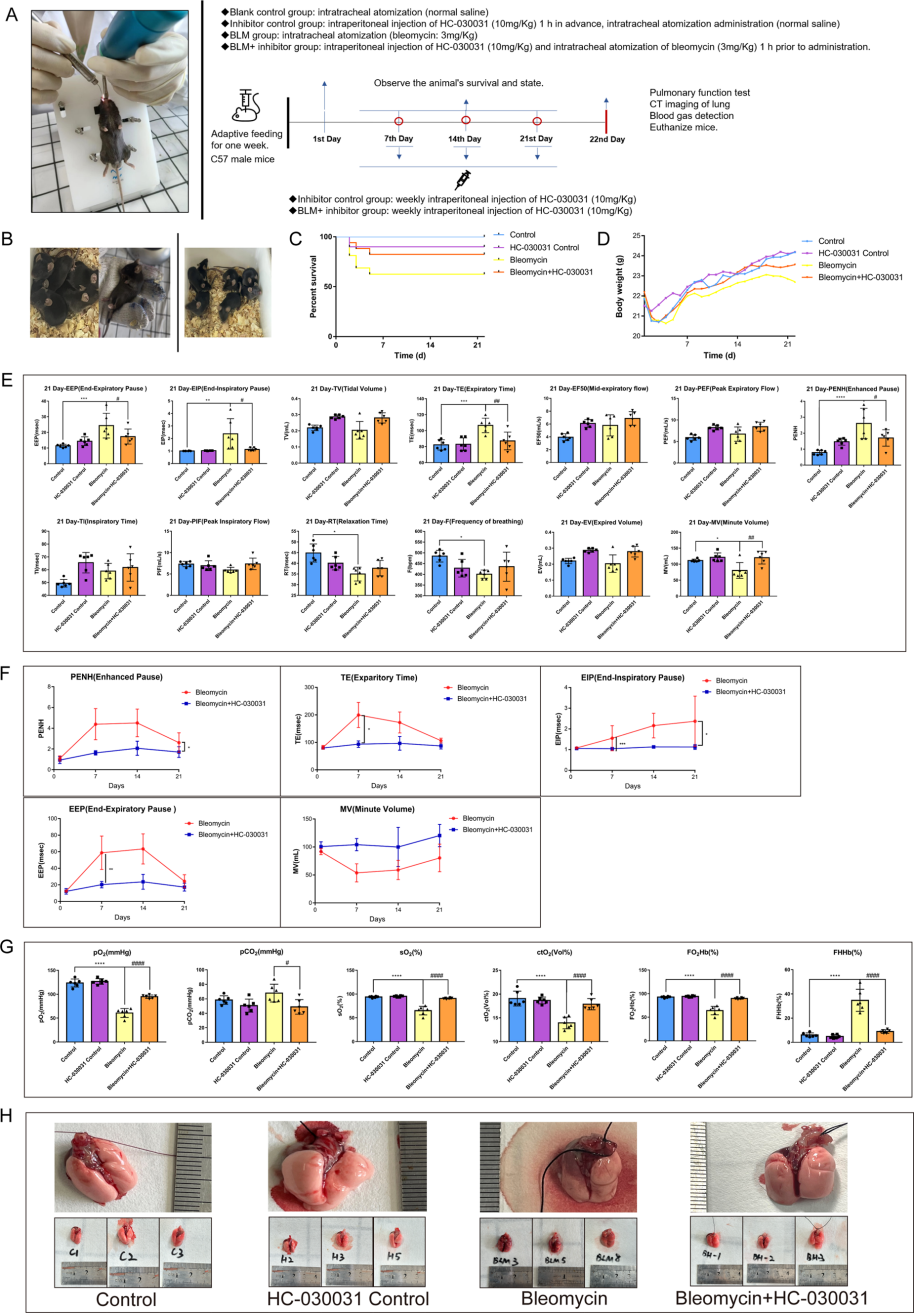
Inhibition of TRPA1 alleviates bleomycin-induced lung injury in mice. **A** Schematic diagram of the establishment of a mouse pulmonary fibrosis model induced by bleomycin. **B** Living status of bleomycin-treated mice. **C** Survival curves of mice. **D** Weight change curve of mice. **E** Lung function test of mice. **F** End-expiratory pressure (EEP), End-inspiratory pressure (EIP), Expiration Time (TE), Enhanced Pause (PENH), Minute volume (MV), and index time change chart. **G** The changed blood gas values of mice. **H** Morphological and anatomical images of mouse lung tissue. Statistical significance was denoted as *P (Bleomycin group vs. Control group, *P < 0.05, **P < 0.01, ***P < 0.001, ****P < 0.0001) and ^#^*P* (Bleomycin + HC-030031 group vs. Bleomycin group, ^#^*P* < 0.05, ^##^*P* < 0.01, ^###^*P* < 0.001, ^####^*P* < 0.0001)

We used aerosolized bleomycin to ensure uniform lung distribution and accurate dosing to establish a pulmonary fibrosis model (Fig. 1A). To further elucidate the role of TRPA1 in bleomycin-induced pulmonary fibrosis, the TRPA1-specific inhibitor, HC-030031, was employed in an intervention experiment. Mice treated with bleomycin showed reduced activity, clustering behavior, and audible breathing, indicating potential respiratory distress or obstruction (Fig. 1B). Additionally, the mice exhibited considerable weight loss, reduced appetite (Fig. 1C), and decreased survival rates (Fig. 1D). However, these behavioral changes, weight loss, and mortality were attenuated by the administration of HC-030031 in mice treated with bleomycin. Lung function was evaluated every seven days. Notable alterations in end-expiratory pause (EEP) and end-inspiratory pause (EIP) were observed, suggesting changes in lung compliance and resistance. Both EEP and EIP were significantly elevated in bleomycin-treated mice compared with those in the control group. However, TRPA1 inhibition significantly attenuated the increases in EEP and EIP. Changes in expiration time (TE) indicate variations in respiratory mechanics, whereas enhanced pauses (PENH), an indicator of airway resistance and airflow limitation, reflect respiratory distress. The experiment showed that TE and PENH were significantly higher in the bleomycin-treated mice than that in the control group, but these increases were reduced after TRPA1 inhibition. Minute volume (MV) changes indicate alterations in the overall lung ventilation capacity. MV in the bleomycin group was significantly lower than that in the control group, but this reduction was alleviated by TRPA1 inhibition (Fig. 1E). These trends, which were monitored every seven days, highlighted impairments in respiratory mechanics and gas exchange following fibrosis. Lung function was severely impaired in bleomycin-treated mice but was ameliorated by treatment with HC-030031 (Fig. 1F). Significant changes were also observed in the blood gas indices. Compared with those of the control mice, bleomycin-treated mice exhibited a significant decrease in the partial pressure of oxygen (pO_2_), increased partial pressure of carbon dioxide (pCO_2_), reduced oxygen saturation (sO_2_), and a marked decrease in total oxygen content (ctO_2_), suggesting impaired blood oxygen-carrying capacity. However, mice treated with HC-030031 showed significant improvements in pO_2_, pCO_2_, sO_2_, and ctO_2_ after exposure to bleomycin. Meanwhile, oxyhemoglobin (FO_2_Hb) decreased and deoxyhemoglobin (FHHb) increased in bleomycin-treated mice, indicating compromised oxygen transport and utilization. However, these trends were reversed by HC-030031 (Fig. 1G). Anatomical examination revealed significant morphological alterations in the lung tissues of bleomycin-treated mice. The previously smooth surfaces of the lungs became red and swollen following bleomycin-induced fibrosis, indicating an inflammatory response and tissue remodeling that characterizes the fibrotic process (Fig. 1H). In brief, this study indicated that mice subjected to bleomycin exposure exhibited increased severity of lung injury and fibrotic lesions, which could be substantially ameliorated by the TRPA1-specific inhibitor, HC-030031.

### TRPA1 inhibition mitigated bleomycin-induced pathological lung injury in mice

**Fig. 2:**
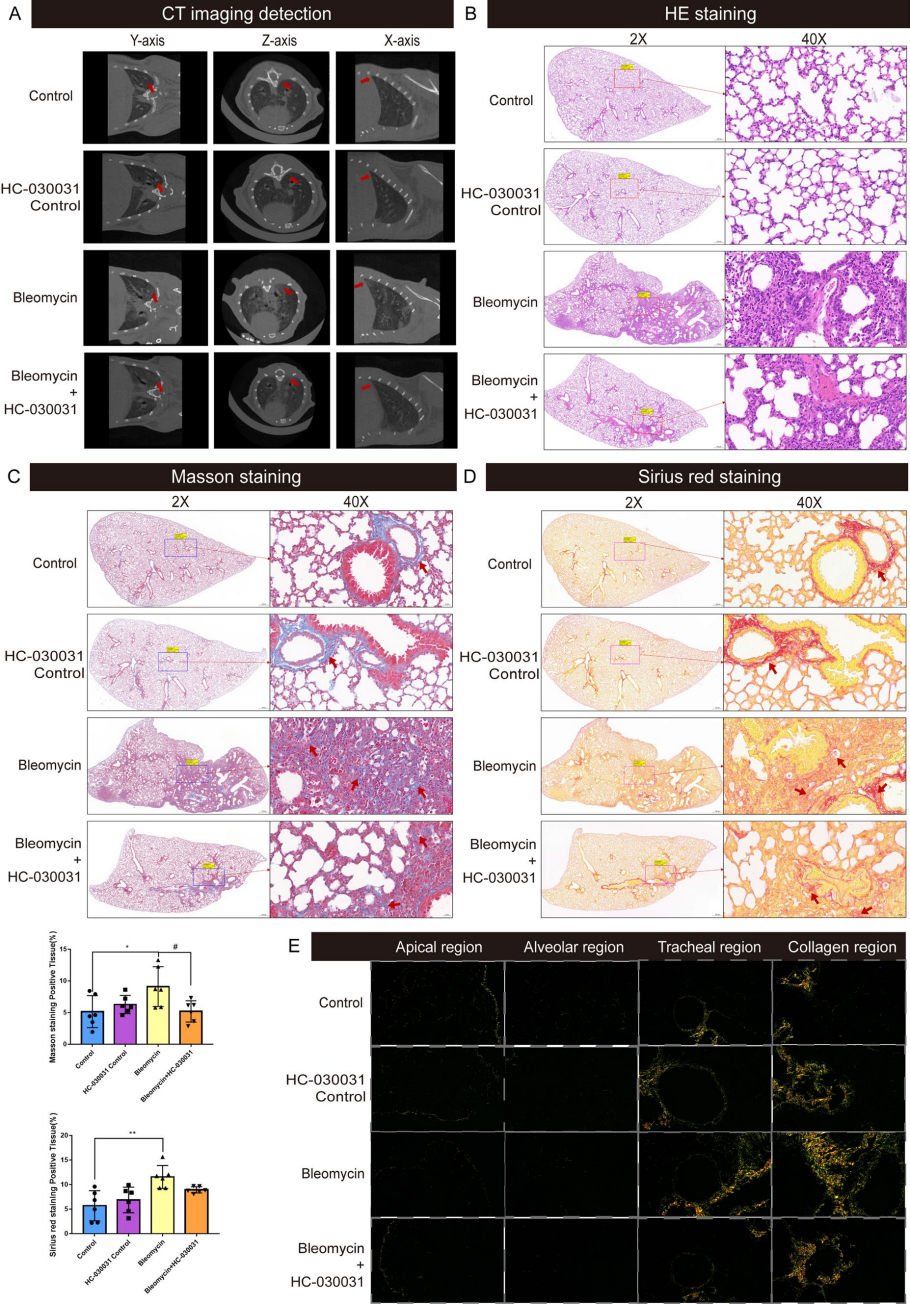
TRPA1 inhibition mitigates bleomycin-induced pulmonary fibrosis in mice. **A** CT images of mouse lung tissue. **B** HE staining: Nuclei are stained dark blue or purple with hematoxylin, whereas eosin imparts various shades of pink and red to the cytoplasm and extracellular matrix. **C** Masson staining: collagen deposition, a hallmark of fibrosis, is indicated by blue staining. **D**, **E** Sirius Red staining: collagen fibers, the primary constituents of fibrotic tissue, are vividly stained in bright red, contrasting distinctly with the surrounding tissue. Figure **B**, **C**, and **D** each include a left column with 2 × magnification for a panoramic view of the lung tissue, and a right column depicting a 40 × magnification for detailed area imaging

CT imaging showed that the apex of the lung was blurred in the bleomycin group, and there were reticular lesions in the lung, while no obvious lesions were found in the control group. Simultaneously, the lesions in the apex of the lung were alleviated after inhibition of TRPA1 (Fig. 2A). HE staining revealed that normal mice displayed thin-walled, well-defined alveoli optimal for gas exchange, whereas bleomycin-treated mice exhibited thickened alveolar walls, disrupted lung structure. Increased stromal cells, and infiltration of fibroblasts and inflammatory cells were observed at the red arrows. Notably, the TRPA1 inhibitor HC-030031 mitigated the structural lung damage and fibrotic tissue replacement (Fig. 2B). Masson staining indicated extensive blue-stained collagen deposits in the bleomycin-treated mice, which surrounded and obliterated the thin alveolar walls. However, as indicated by the arrow, the TRPA1 inhibitor HC-030031 significantly reduced the area of collagen deposition, and normal pink myofibers and cytoplasm were restored in most areas of the lung tissue, with only a small amount of blue fiber deposition visible around the airways (Figs. 2C, S2). Sirius Red staining showed dark red-stained collagen fiber areas in the bleomycin-treated mice, contrasting sharply with the surrounding tissue and disrupted the normal lung structure. Visible at the arrow mark, the TRPA1 inhibitor HC-030031 attenuated alveolar and bronchiolar damage and obstruction caused by collagen deposition (Figs. 2D, S3). Using polarized light microscopy on the Sirius Red-stained sections of the apical region, alveolar region, trachea region, and collagen deposition region, we found that the bleomycin-treated mice infected through their trachea, exhibited strong yellow refraction in the collagen deposition region. This indicates a high concentration of type I collagen, which may provide valuable insights for subsequent molecular tests (Fig. 2E).

### Inhibition of TRPA1 significantly alleviated the enhanced Young’s modulus in lung tissue following fibrosis

**Fig. 3:**
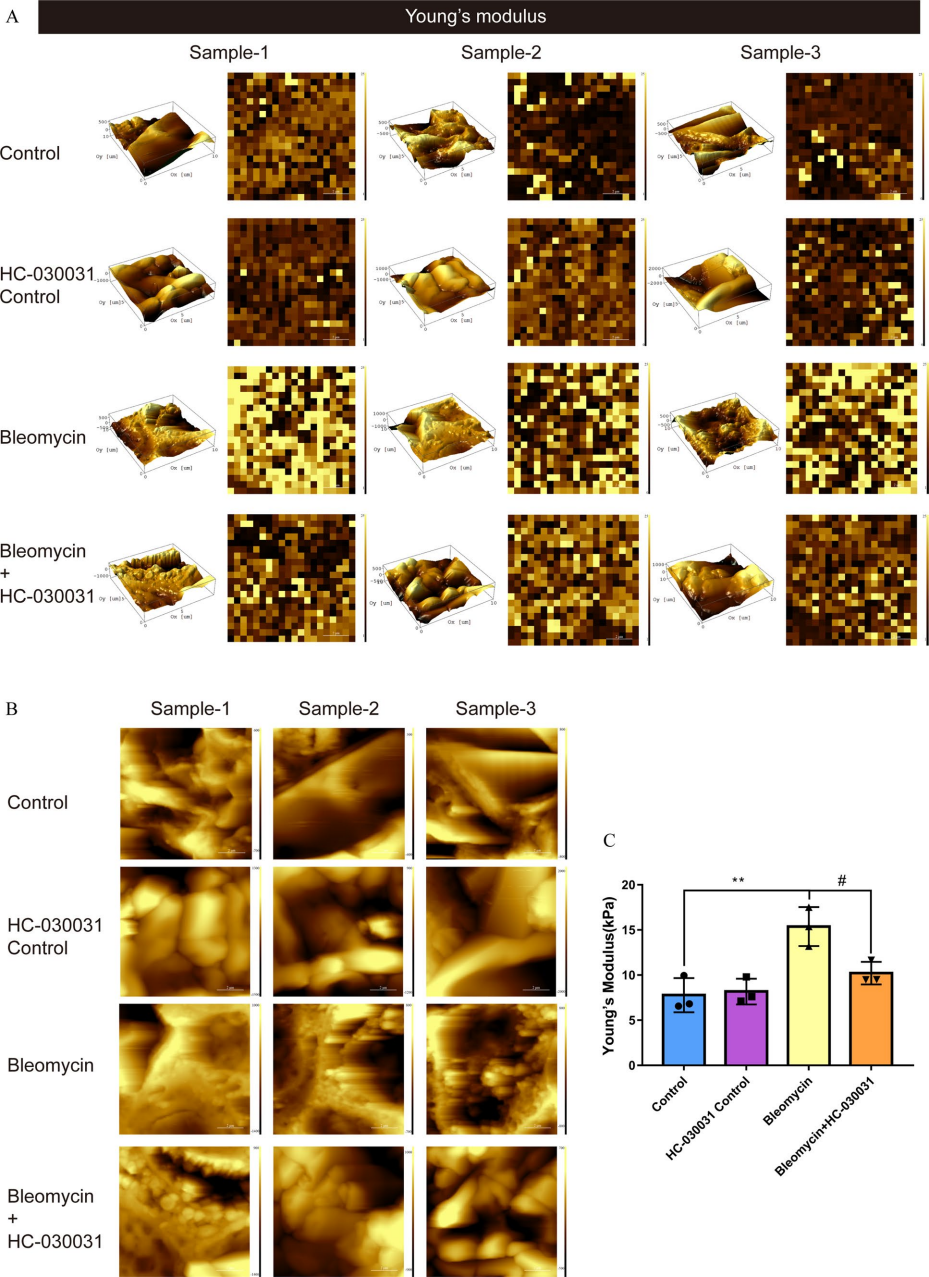
TRPA1 inhibition significantly attenuates the increase in Young's modulus of lung tissue after fibrosis.** A** Three-dimensional (3D) imaging of lung tissue using atomic force microscopy (AFM). **B** Topography of lung tissue. **C** Three lung tissues were randomly selected from each group, and the mean values of 10 points in each lung tissue were analyzed to compare the elastic modulus of lung tissue of mice exposed to bleomycin. The results were expressed as Young's modulus (kPa). Statistical significance was denoted as *P (Bleomycin group vs. Control group, *P < 0.05, **P < 0.01, ***P < 0.001, ****P < 0.0001) and ^#^*P* (Bleomycin + HC-030031 group vs. Bleomycin group, ^*#*^*P* < 0.05, ^##^*P* < 0.01, ^###^*P* < 0.001, ^####^*P* < 0.0001)

The inhibition of TRPA1 significantly mitigated the increase in lung tissue Young’s modulus, a hallmark of fibrotic stiffening. Changes in alveolar tissue shape after fibrosis were observed in images captured and analyzed using AFM, and HC-030031 mitigated this effect (Fig. 3A, B). Following treatment with a TRPA1 inhibitor, AFM revealed a marked reduction in the elastic modulus of lung tissues from mice subjected to bleomycin-induced fibrosis, compared with that of untreated controls (Fig. 3C). This reduction in stiffness indicates a lessened fibrotic burden, suggesting that TRPA1 plays a crucial role in the fibrogenic process that leads to the hardening of lung tissue. Therefore, TRPA1 inhibition can effectively alleviate the mechanical alterations associated with pulmonary fibrosis.

### TRPA1 inhibition attenuated pro-fibrotic marker expression and collagen deposition in bleomycin-induced pulmonary fibrosis in mice

**Fig. 4:**
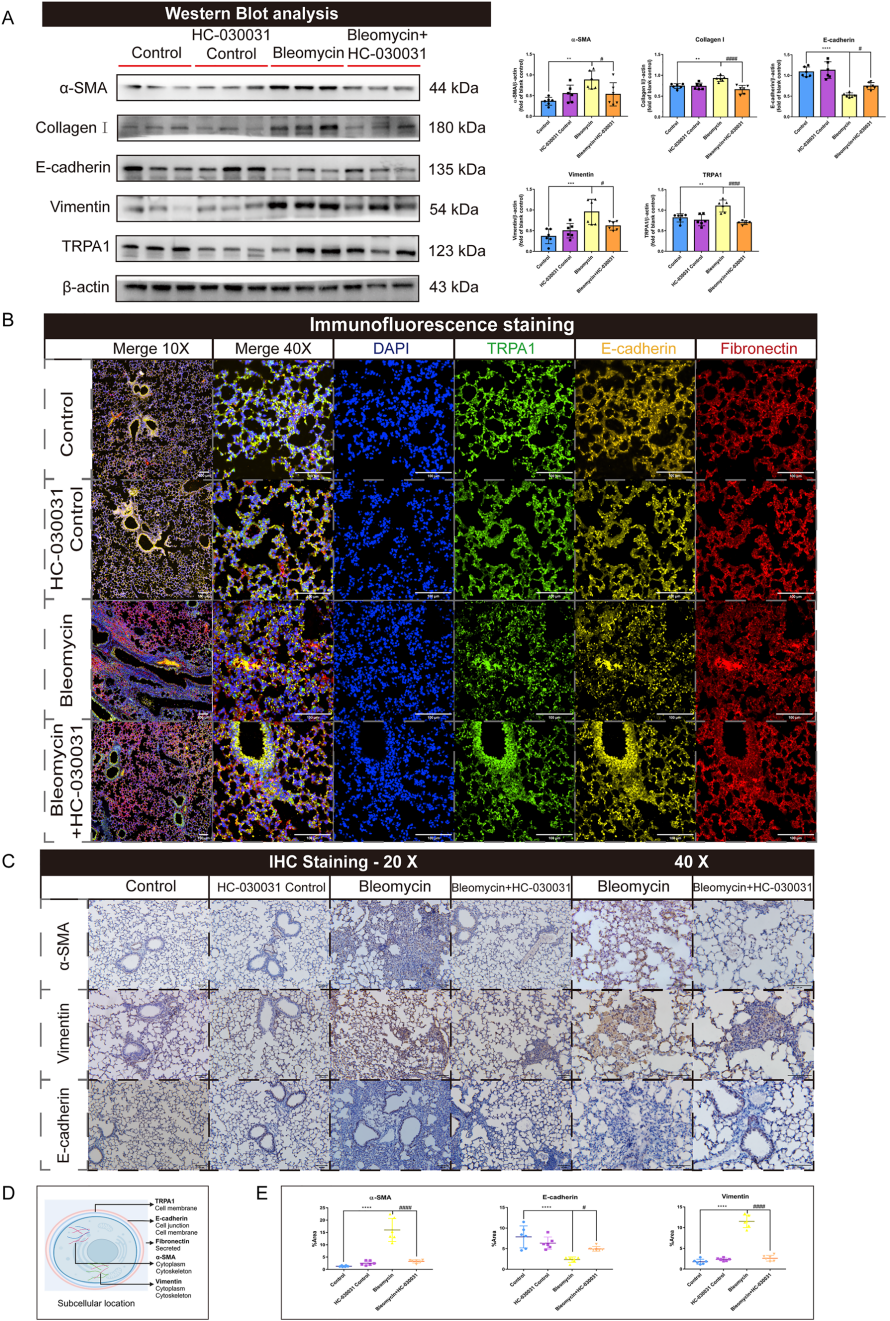
Inhibition of TRPA1 attenuates bleomycin-induced collagen deposition and profibrotic marker expression in mouse lung tissues. **A** Western blot analysis showed significant changes in fibrosis-associated protein (α- SMA, collagen Ι, E-cadherin, and vimentin) and TRPA1 expression. **B** Immunofluorescence staining: Nuclei were stained with DAPI, exhibiting blue fluorescence. TRPA1 was labeled with FITC, showing green fluorescence. E-cadherin in the Cy3 channel emitted yellow fluorescence, and fibronectin in the Cy5 channel displayed red fluorescence. Merge 10 × and Merge 40 × images represent composite views of all four channels at 10 × and 40 × magnification, respectively. **C** The protein expressions of α-SMA, vimentin, and E-cadherin were detected using immunohistochemical staining. **D** Schematic representation of the cellular localization of TRPA1, E-cadherin, fibronectin, α-SMA, and vimentin. **E** The expression of α-SMA, vimentin, and E-cadherin was detected using immunohistochemistry. Statistical significance was denoted as *P (Bleomycin group vs. Control group, *P < 0.05, **P < 0.01, ***P < 0.001, ****P < 0.0001) and ^#^*P* (Bleomycin + HC-030031 group vs. Bleomycin group, ^*#*^*P* < 0.05, ^##^*P* < 0.01, ^###^*P* < 0.001, ^####^*P* < 0.0001)

Western blot analysis was conducted to assess the expression of the fibrosis-associated proteins. The results showed that α-SMA and collagen I, markers of myofibroblast activation and collagen deposition, respectively, were significantly elevated in the pulmonary tissue of bleomycin-treated mice compared with those in the control group. In contrast, mice treated with HC-030031, a TRPA1 inhibitor, exhibited a significant decrease in the levels of α-SMA and collagen I compared with those in bleomycin-treated mice. Furthermore, bleomycin-treated mice showed decreased expression of E-cadherin, an indicator of epithelial integrity, suggesting epithelial-mesenchymal transition (EMT) had occurred. However, TRPA1 inhibition resulted in increased E-cadherin expression. Vimentin, a mesenchymal marker, was also upregulated in bleomycin-treated mice, supporting the occurrence of EMT; however, its expression was decreased after HC-030031 treatment. Concurrently, TRPA1 expression was significantly increased in bleomycin-treated mice compared with that in untreated mice, indicating its potential involvement in fibrosis pathogenesis (Fig. 4A). Immunofluorescence analysis also confirmed a significant increase in TRPA1 (FITC, green) and fibronectin (Cy5, red) expression, as well as decreased E-cadherin (Cy3, yellow) expression in the pulmonary tissue of bleomycin-treated mice, indicating enhanced extracellular matrix deposition and decreased epithelial integrity, however, this profibrotic process was alleviated when TRPA1 was inhibited (Fig. 4B). Immunohistochemical staining also revealed significant alterations in α-SMA, vimentin, and E-cadherin. The localization and quantity of α-SMA and vimentin were increased in the fibrotic regions of bleomycin-treated mice compared with those in the control group. However, HC-030031 treatment significantly decreased the expression of α-SMA and vimentin compared with that in bleomycin-treated mice. Meanwhile, E-cadherin was also significantly reduced in bleomycin-treated mice due to EMT, and its expression was restored upon TRPA1 inhibition (Fig. 4C, D and E). These findings suggest that TRPA1 is involved in lung tissue fibrosis and cellular dynamics.

### Inhibition of TRPA1 reduced M2 macrophages polarization

**Fig. 5:**
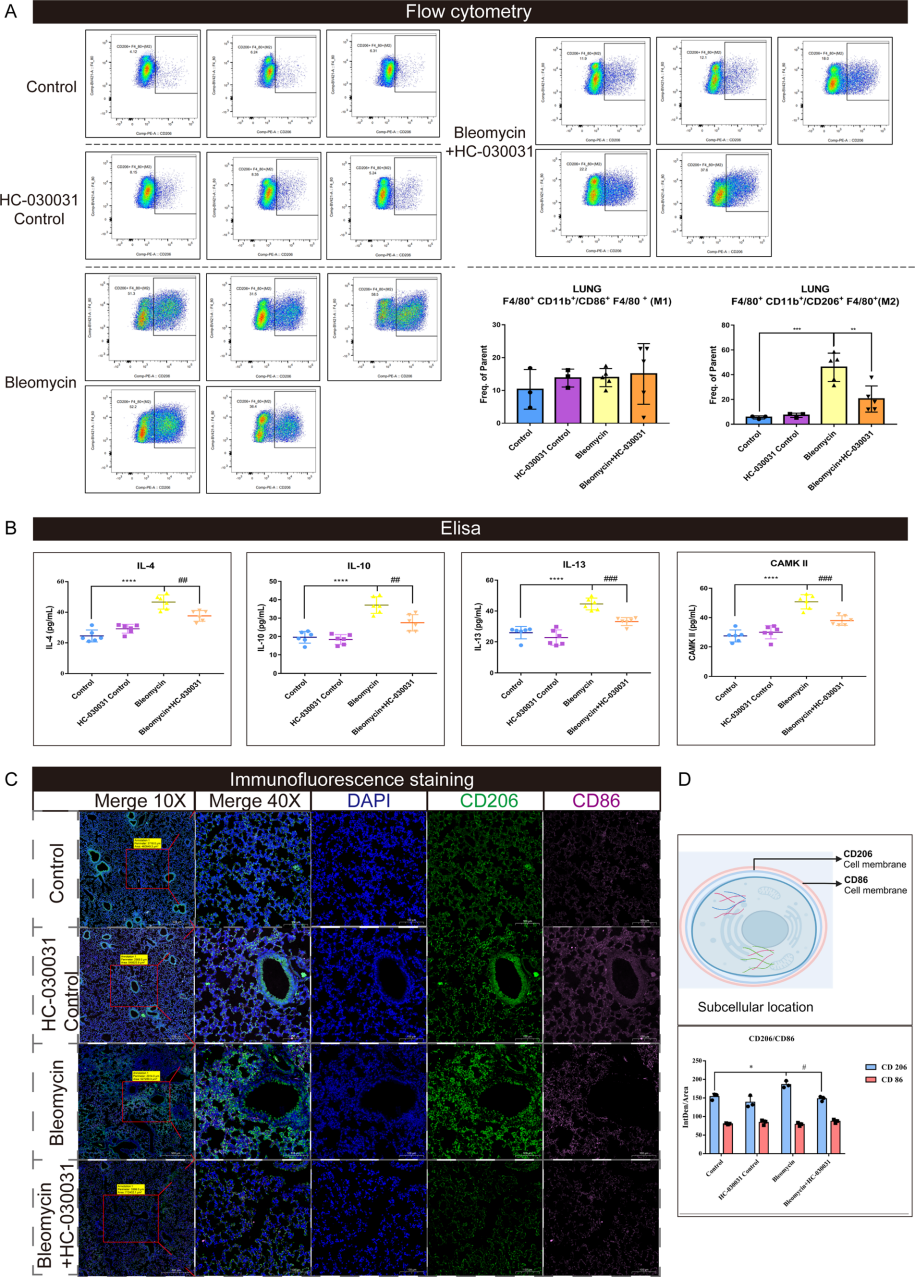
Inhibition of TRPA1 reduces M2 macrophage polarization. **A** Flow cytometry was used to identify alveolar macrophages (F4/80^+^&CD11b^+^), M2-type (F4/80^+^&CD206^+^), and M1-type (F4/80^+^&CD86^+^) macrophages. **B** ELISA was performed on mouse serum to measure IL-4, IL-10, IL-13, and CaMKII levels. **C** Immunofluorescence staining: DAPI stained the nuclei blue, FITC labeled CD206 displayed green fluorescence, and CD86 in the Cy3 channel appeared pink. Merged 10 × and 40 × images provide composite views at respective magnifications. **D** Schematic representation of cellular localization and quantification of CD206 and CD86. Statistical significance was denoted as *P (Bleomycin group vs. Control group, *P < 0.05, **P < 0.01, ***P < 0.001, ****P < 0.0001) and ^#^*P* (Bleomycin + HC-030031 group vs. Bleomycin group, ^#^*P* < 0.05, ^##^*P* < 0.01, ^###^*P* < 0.001, ^####^*P* < 0.0001)

The macrophage polarization in mouse lung tissue was analyzed using flow cytometry. Macrophages were labeled as F4/80^+^ and CD11b^+^. In M2 polarization, CD206^+^ expression increases, whereas CD86^+^ expression increases in M1 polarization. The results showed that the number of CD206^+^ cells in the lung tissue of bleomycin-treated mice increased significantly compared with that in the control group, indicating a shift towards M2 polarization. However, mice treated with HC-030031 showed a decrease in CD206^+^ cells. Simultaneously, there was no significant difference in the CD86^+^ cells, a marker for M1 polarization, among the groups (Fig. 5A). Additionally, cytokine levels in mouse serum were evaluated using ELISA. Bleomycin-treated mice showed significant increases in serum IL-4, IL-10, and IL-13 levels, indicating an enhanced systemic inflammatory response and immune regulation associated with fibrosis. Simultaneously, the expression of calmodulin-dependent protein kinase (CaMK)II, a calmodulin-dependent kinase representing calcium ions, increased significantly. However, HC-030031 decreased IL-4, IL-10, IL-13, and CaMKII serum levels compared with those in bleomycin-treated mice (Fig. 5B). These findings suggest that TRPA1 inhibition may disrupt macrophage polarization towards the M2 phenotype, given the key roles of these cytokines in immune modulation and M2 macrophage polarization. Immunofluorescence staining revealed changes in CD206 and CD86 expression in lung tissue. CD206 (FITC, green) expression increased, particularly in fibrotic areas, but was attenuated by treatment with the TRPA1 inhibitor, HC-030031. However, CD86 (Cy3, pink), marking M1-type macrophages, exhibited minimal variation across the groups (Fig. 5C). Colocalization and quantitative analyses underscored the shift towards M2-type polarization in lung fibrosis (Fig. 5D).

### Inhibition of TRPA1 attenuated M2 macrophage polarization by suppressing phosphorylation in the TGF-β1-Smad2 pathway

**Fig. 6:**
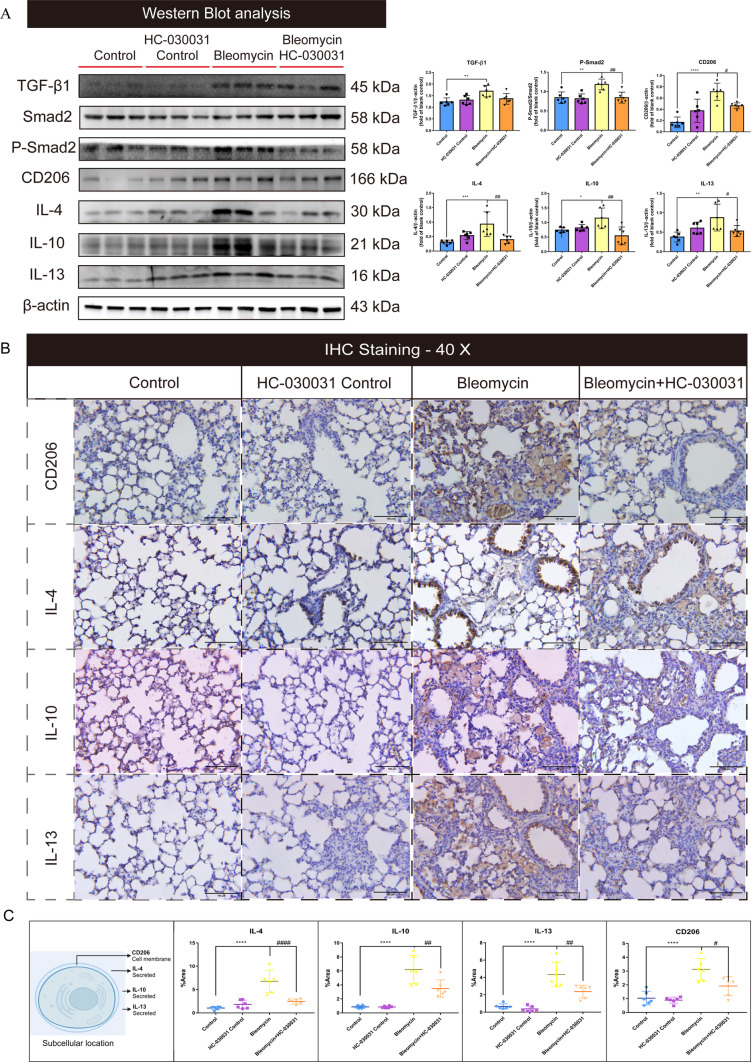
Inhibition of TRPA1 diminishes macrophage polarization to the M2 phenotype by disrupting the phosphorylation of the TGF-β1-suppressor of Smad2 pathway. **A** Western blot analysis of TGF-β1-Smad2 pathway activation and the expression of macrophage polarization markers CD206, IL-4, IL-10, and IL-13. **B** Immunohistochemical staining to detect the expression of CD206, IL-4, IL-10, and IL-13 proteins. **C** Schematic representation of cellular localization and quantification of CD206, IL-4, IL-10, and IL-13. Statistical significance was denoted as *P (Bleomycin group vs. Control group, *P < 0.05, **P < 0.01, ***P < 0.001, ****P < 0.0001) and ^#^*P* (Bleomycin + HC-030031 group vs. Bleomycin group, ^#^*P* < 0.05, ^##^*P* < 0.01, ^###^*P* < 0.001, ^####^*P* < 0.0001)

Our investigation then honed in on the link between TRPA1 and the polarization of M2 macrophages, with a particular emphasis on the TGF-β1-Smad2 signaling pathway, recognized as a pivotal regulator of macrophage polarization [23–25]. Western blot analysis revealed that bleomycin-exposed mice exhibited a significant increase in TGF-β1 expression compared with that in the control group. Additionally, there was enhanced phosphorylation of the downstream Smad2/p-Smad2 pathway in these mice. Interestingly, HC-030031 did not observably change the expression of TGF-β1, but significantly reduced Smad2 phosphorylation, suggesting that TRPA1 inhibition affects Smad2 phosphorylation (Fig. 6A). Furthermore, we found that the expression levels of CD206, IL-4, IL-10, and IL-13 in bleomycin-treated mice were significantly increased compared with those in the control group, and the TGF-β1-Smad2 pathway could regulate macrophages polarization to the M2 type through TRPA1 inhibitor intervention. However, the CD206, IL-4, IL-10, and IL-13 levels were significantly downregulated by HC-030031 compared with those in bleomycin-treated mice. In addition, immunohistochemical staining also revealed a significant increase in CD206 expression in fibrotic areas, indicating enhanced M2-type macrophage activation. Similarly, elevated levels of IL-4, IL-10, and IL-13 were observed, which was consistent with M2 macrophage functions in immune regulation and tissue remodeling. Notably, the TRPA1 inhibitor HC-030031 significantly decreased the expression of CD206, IL-4, IL-10, and IL-13 compared with those in the bleomycin-treated mice (Fig. 6B, C). These findings highlight an important role of M2-phenotype macrophages and their related cytokines in the progression of pulmonary fibrosis, and the TRPA1 inhibitor prevents the transition of macrophages to the M2 phenotype. Meanwhile, the inhibitory effect of TRPA1 inhibitors on M2 polarization may be regulated by inhibiting the phosphorylation of Smad2 pathway downstream of TGF-β1.

### Inhibition of TRPA1 effectively impeded the bleomycin-induced M2 polarization of THP-1 cells

**Fig. 7:**
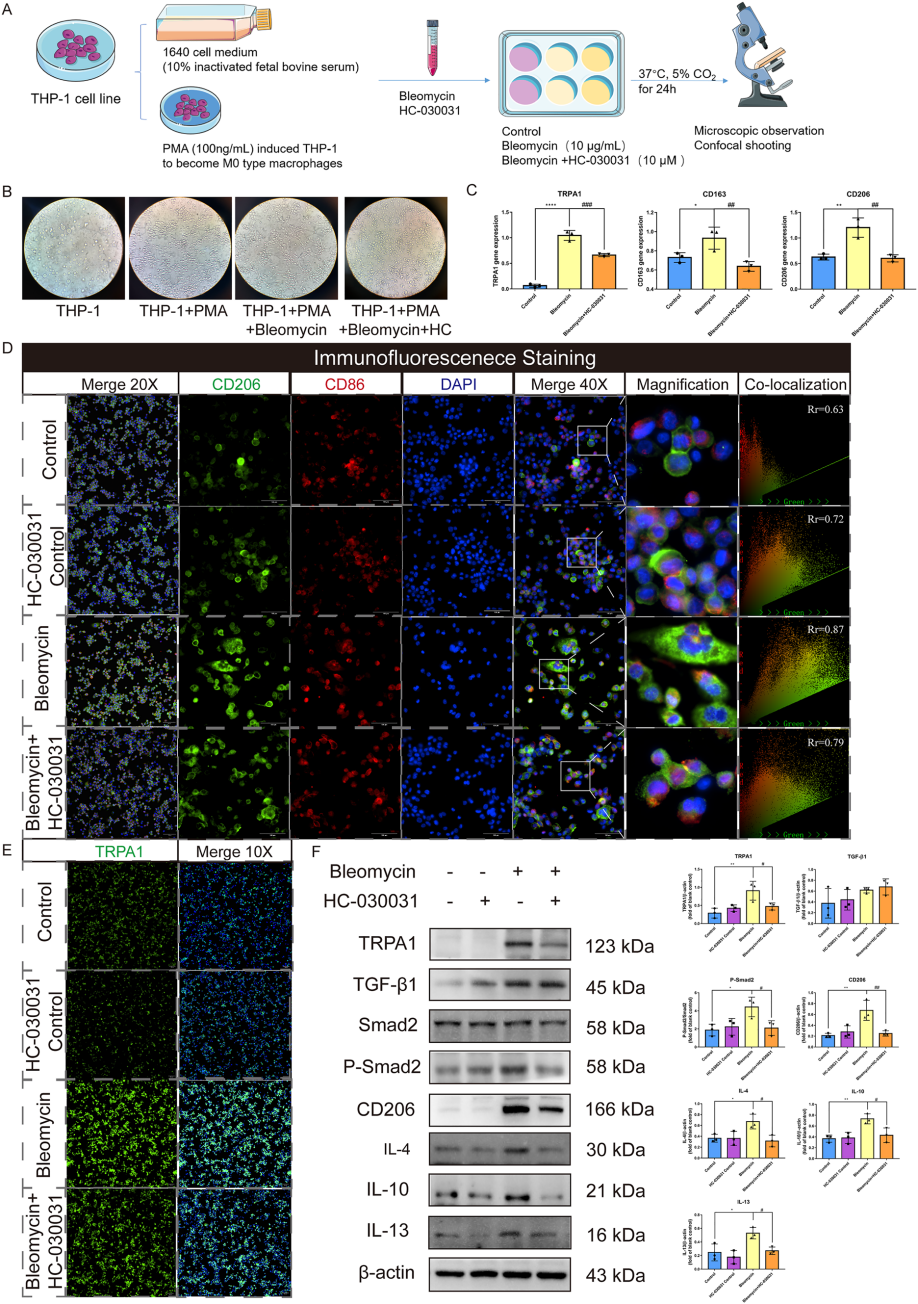
Inhibition of TRPA1 attenuates bleomycin-induced polarization of THP-1 cells toward the M2 phenotype. **A** THP-1 cells were induced into macrophages using PMA, and bleomycin was used to stimulate macrophage polarization. **B** Morphological changes of THP-1 cells under microscope. **C** TRPA1, CD206 and CD163 mRNA were detected using RT-qPCR. **D** Immunofluorescence staining: DAPI stains the nucleus blue, FITC-labeled CD206 in green fluorescence, and CD86 in Cy5 channel in red. The combined 20 × and 40 × images provide a composite view at their respective magnifications. Scatter plots of regional magnification show colocalization and expression of CD206 and CD86. **E** Immunofluorescence staining: DAPI stains the nucleus blue, and FITC-labeled TRPA1 shows green fluorescence. **F** Western blot to detect the activation of TRPA1 and TGF-β1-Smad2 pathways and the expression of macrophage polarization markers CD206, IL-4, IL-10, and IL-13. Statistical significance was denoted as *P (Bleomycin group vs. Control group, *P < 0.05, **P < 0.01, ***P < 0.001, ****P < 0.0001) and ^#^*P* (Bleomycin + HC-030031 group vs. Bleomycin group, ^#^*P* < 0.05, ^##^*P* < 0.01, ^###^*P* < 0.001, ^####^*P* < 0.0001)

Next, THP-1 cells were used to establish an in vitro macrophage polarization model. First, THP-1 cells were induced to differentiate into macrophages using phorbol 12-myristate 13-acetate (PMA), and then stimulated with bleomycin to detect changes in their polarization indicators (Fig. 7A). In vitro, we found that THP-1 cells differentiated into M0 macrophages in response to PMA (Fig. 7B). This was evident in the morphological changes observed, where the cells transformed from a monocyte-like appearance to a large adherent phenotype with distinct cytoplasmic extensions. However, bleomycin + HC-030031-treated THP-1 cells showed fewer morphological changes than bleomycin-treated cells did. TRPA1, CD206, and CD163 mRNA levels were also detected using RT-qPCR after bleomycin stimulation (Fig. 7C). We found that bleomycin-treated cells showed increased expression of TRPA1, CD206, and CD163 compared with that in control cells, whereas TRPA1, CD206, and CD163 expression was significantly decreased in THP-1 cells treated with HC-030031. Immunofluorescence analysis demonstrated that CD206 expression was enhanced in bleomycin-treated cells compared with that in the control group; however, it was significantly decreased by HC-030031. The 40 × composite plot showed that CD206 and CD86 were mostly expressed in the cell membrane. The co-localization analysis of the magnified region showed that the co-expression areas of CD206 and CD86 in the control and bleomycin-treated cells were low, the Rr value was low, the macrophages were polarized in a certain direction, and the green fluorescence of CD206 in bleomycin-treated cells was strong. No significant difference was observed in the fluorescence intensity of CD86 between the control and bleomycin-treated THP-1 cells. Colocalization analysis provided insights into the intracellular distribution and interaction of these markers, highlighting the transition to the M2 phenotype after bleomycin induction (Fig. 7D). Immunofluorescence analysis showed that the fluorescence intensity of TRPA1 in bleomycin-treated cells was significantly enhanced compared with that in the control group, which was significantly reduced after intervention with HC-030031 (Fig. 7E). Furthermore, western blot revealed that TRPA1 expression was significantly upregulated in bleomycin-treated cells, whereas it was significantly decreased after HC-030031 intervention. The expression of TGF-β1 was increased in bleomycin-treated cells, and the phosphorylation of the downstream Smad2/P-Smad2 pathway was also enhanced. However, HC-030031 didn’t obviously change the TGF-β1 expression in bleomycin-treated cells compared with that in the control group but significantly decreased the expression of Smad2 phosphorylation. Meanwhile, the levels of CD206, IL-4, IL-10, and IL-13 in bleomycin-treated cells were significantly increased compared with that in the control group. However, HC-030031 significantly decreased the expression of CD206, IL-4, IL-10, and IL-13 (Fig. 7F). These findings suggest that TRPA1 inhibition downregulates M2 macrophages polarization, potentially by interfering with the phosphorylation of the Smad2 pathway in vitro.

## Discussion

IPF is a chronic fibrotic lung disease characterized by dry cough, fatigue, and progressive exertional dyspnea. The disease pathologically involves the destruction of the lung parenchyma and architecture, leading to loss of compliance and compromised gas exchange. This debilitating condition often results in respiratory failure and death within 3–5 years of diagnosis [26]. The etiopathogenesis of IPF remains unclear, thereby complicating the development of effective treatments. The pathological features of this disease include extracellular matrix remodeling, fibroblast activation and proliferation, immune dysregulation, cell senescence, and the presence of aberrant basaloid cells [27, 28]. Currently, the mainstay therapies for IPF include oral anti-fibrotic drugs such as pirfenidone and nintedanib [29, 30]. These treatments have shown efficacy in improving quality of life, attenuating symptoms, and slowing disease progression. However, to date, unilateral or bilateral lung transplantation remains the only treatment option proven to increase the life expectancy of patients with IPF. Given these challenges, accelerated research into the mechanisms underlying IPF is urgently necessary. Understanding these mechanisms is crucial for developing effective therapeutics to increase life expectancy, alleviate symptoms, and improve the overall well-being of patients with IPF [26].

TRPA1 channels, part of the TRP channel superfamily, play crucial roles in various physiological processes, including acting as nonselective cation channels permeable to Ca^2+^ [31]. Several chemicals, including allyl isothiocyanate, activate these channels and have been implicated in fibrotic processes, including cardiac fibrosis [32]. Oxides and other chemical stimuli also activate TRPA1 [33, 34]. The activation of TRPA1 is closely associated with oxidative stress and the inflammatory response [35, 36] while oxidative stress and inflammation are the main drivers of disease progression [37]. The involvement of TRPA1 in pulmonary fibrosis has garnered significant interest. Our research marks the first to show a marked activation of TRPA1 in a bleomycin-induced mouse model of pulmonary fibrosis. Utilizing HC-030031, a TRPA1-specific inhibitor, we explored its effects on this condition. Mice treated with bleomycin displayed symptoms of lung damage such as reduced activity, weight loss, and diminished survival, alongside impaired lung function, including lowered compliance and ventilation capacity, and increased airway resistance. Blood tests indicated decreased oxygen levels, while CT scans and lung examinations revealed extensive fibrotic changes and structural damage. Treatment with HC-030031 reduced these symptoms, as seen in lower fibroblast and inflammatory cell presence, reduced collagen buildup, and improved lung architecture on pathological staining. Western blot and immunohistochemical analyses showed decreased fibrosis markers and partial recovery of cellular integrity, underscoring TRPA1's potential as a target for mitigating lung fibrosis.

In contrast, the increase of Young's modulus after lung tissue fibrosis represents the decrease of lung compliance with excessive tissue repair and collagen deposition. These results suggest that the tissue repair function of macrophages may play a key role in the regulation of pulmonary fibrosis [38–40]. Due to their plasticity, macrophages differentiate into various phenotypes, including M1 and M2 macrophages, in response to the pulmonary fibrosis microenvironment [41]. In the early stages of pulmonary fibrosis, an increase in the number of classically activated M1 macrophages helps clear pathogens and promotes inflammation. However, during fibrosis progression, alternatively activated M2 macrophages, which inhibit inflammation or directly promote tissue fibrosis, are predominant [42]. Further studies have highlighted that the M1 and M2 macrophage phenotypes correspond to pro-inflammatory and pro-fibrogenic genic signatures, respectively [43]. Particularly, M2 macrophages have been shown to promote myofibroblast differentiation, which is a key aspect of pulmonary fibrogenesis [44]. The blockade of pulmonary macrophage infiltration attenuates bleomycin-induced pulmonary fibrosis, underscoring the critical role of M2 macrophages in this disease's pathogenesis [44]. Recently, TRPA1 activation induced macrophage polarization to the M2 phenotype [45], and we further explored its role in pulmonary fibrosis. In our study, TRPA1 inhibition reduced macrophage polarization towards the M2 phenotype in a mouse model of pulmonary fibrosis. Flow cytometry analysis showed that TRPA1 inhibition decreased CD206^+^ M2 macrophages in lung tissue. ELISA results indicated reduced levels of M2-associated cytokines (IL-4, IL-10, and IL-13) in mice treated with HC-030031 compared with those in mice treated with bleomycin, suggesting that TRPA1 inhibition disrupted M2 macrophage polarization and immune modulation.

In addition, pulmonary fibrosis is a complex disease, and its pathological processes involve the interaction of multiple cell types and signaling pathways. The TGF-β1 and Smad2 pathways play key roles in regulating pulmonary fibrosis [46]. TGF-β1 is a multifunctional growth factor that has been extensively studied and is associated with a variety of cellular functions and pathological processes [47, 48]. In pulmonary fibrosis, the expression of TGF-β1 usually increases, especially in the damaged lung tissue. TGF-β1 can induce the polarization of macrophages to the M2 phenotype, a macrophage subtype closely associated with pulmonary fibrosis [49, 50]. This process involves a series of signaling pathways, of which the Smad2 pathway is a key component [51, 52]. Among them, pirfenidone regulates macrophage polarization and improves radiation pulmonary fibrosis by inhibiting the TGF-β1/Smad2/3 pathway [53]. Given the role of TRPA1 in fibrosis, its deficiency or inhibition could have therapeutic effects on diseases such as pulmonary fibrosis. This assumption is supported by evidence of reduced fibrosis in TRPA1-deficient mice in models of scleroderma dermal fibrosis, cardiac hypertrophy, fibrosis, and corneal stroma healing [18–20]. Our study demonstrated that inhibiting TRPA1 dampened the shift towards M2 macrophage polarization in a mouse model of pulmonary fibrosis, primarily by blocking the TGF-β1-Smad2 pathway's phosphorylation process. Western blot results indicated that TRPA1 blockade reduced Smad2 phosphorylation, hinting at its regulatory role in macrophage polarization. Immunohistochemistry showed decreased M2 macrophage activation and related cytokine levels in fibrotic regions upon TRPA1 inhibition, highlighting the significance of M2 macrophages and the TGF-β1-Smad2 pathway in the disease's progression. Additionally, experiments with THP-1 cells showed that TRPA1, along with M2 markers CD206 and CD163, were upregulated after bleomycin exposure but decreased following HC-030031 treatment. Immunofluorescence and Western blot analyses confirmed these findings, underscoring TRPA1 inhibition's potential to mitigate M2 polarization and interfere with the TGF-β1-Smad2 pathway, offering a promising direction for pulmonary fibrosis treatment.

Exploring the connection between TRPA1 channels and M2 macrophage polarization in IPF progression presents a fascinating research frontier. The paradoxical role of TRPA1 in fibrosis, especially its complex interactions with TGF-β1 across various tissues and pathological conditions, is a critical focus of current studies. In ocular fibroblasts, the loss of TRPA1 attenuates TGF-β1 signaling, thereby reducing inflammatory cytokine expression and myofibroblast trans-differentiation, indicating an anti-fibrotic effect [20]. However, TRPA1 activation can counteract TGF-β1-induced fibrotic changes in bladder sub-urothelial myofibroblasts, suggesting its potential anti-fibrotic role [54]. In models of cardiac hypertrophy and fibrosis, increased TRPA1 expression in failing human hearts and hypertrophic mouse hearts suggests its involvement in cardiac hypertrophy. Suppressing TRPA1 can mitigate fibrosis, shown by reduced levels of fibrotic markers like connective tissue growth factor and collagens I and III [19]. Aligning with our findings, TRPA1 inhibition was found to decrease macrophage infiltration in cardiac tissue, notably curbing M2-phenotype macrophage polarization critical to cardiac fibrosis, thus impacting inflammatory and fibrotic pathways.

Furthermore, in discussing the paradoxical role of TRPA1 in lung fibrosis and its complex relationship with TGF-β1, the literature presents intriguing insights. TRPA1, which is primarily expressed in neuronal and non-neuronal cells such as fibroblasts, plays diverse roles in fibrosis. In pulmonary diseases, TRPA1 activation leads to increased release of IL-8 and matrix metalloproteinase 9 gene expression in lung fibroblasts, suggesting a pro-fibrotic role [55]. However, the role of TRPA1 in fibrosis is not straightforward, as it can also exert anti-fibrotic effects. In lung fibroblasts, TRPA1 expression was downregulated by TGF-β1, affecting myofibroblast survival and resistance to cell death [56]. This is highlighted by its ability to modulate TGF-β1 signaling pathways. Specifically, Ca^2+^ influx through TRPA1 channels leads to extracellular signal-regulated kinase 1/2 phosphorylation, which induces SMAD2 phosphorylation [57]. This type of SMAD2 phosphorylation counteracts pro-fibrotic TGF-β1 signaling by reducing the transcription of profibrotic genes. Our study also found that TRPA1 could regulate phosphorylation of TGF-β1-Smad2 pathways, thereby playing a key role in M2 phenotype polarization.

TRPA1 channels, as highlighted in our study, stand as a complex entity within the fibrotic milieu, possessing the dual capacity to both exacerbate and ameliorate fibrotic processes, contingent upon the specific pathways and contextual nuances at play—echoing the sentiment that “variety is the spice of life” in the realm of pulmonary fibrosis research [58]. To reveal the complex role of TRPA1 in pulmonary fibrosis and macrophage differentiation, we carefully designed in vivo and in vitro experiments to capture models at different time points and dissect their pathological changes. However, our study has several limitations that warrant further investigation. Notably, the absence of a broader range of interventional strategies limits the depth of our exploration into the role of TRPA1. Additionally, the lack of primary macrophage isolation for in vitro experiments restricts our ability to provide a more detailed insight into the role of macrophage polarization shifts in pulmonary fibrosis. Acknowledging these shortcomings, we plan to develop TRPA1 knockout mouse models and observe the effects at various time points to gain a more comprehensive understanding of the temporal dynamics of TRPA1 involvement in pulmonary fibrosis. We intend to perform transcriptome sequencing at different stages of the disease to elucidate the signaling pathways associated with TRPA1 more clearly. Furthermore, we will isolate macrophages for in vitro validation experiments to delve deeper into the molecular mechanisms of macrophage polarization and its role in the pathogenesis of pulmonary fibrosis.

Taken together, our findings highlight the critical role of TRPA1 channels in the pathogenesis of IPF by promoting the phosphorylation of TGF-β1-Smad2 signaling and regulating the polarization of macrophages to the M2 phenotype, which further promotes tissue repair and collagen deposition. This foundational understanding sets the stage for subsequent investigations into the regulatory dynamics of TRPA1 in fibrotic conditions, which is anticipated to enrich our comprehension of pulmonary fibrosis pathogenesis and inspire the development of targeted therapeutic strategies in forthcoming research initiatives.

### Supplementary Information

Below is the link to the electronic supplementary material.

Figure S1: TRPA1 expression is increased in mice of bleomycin-induced pulmonary fibrosis. (A) Western blot analysis showed significant changes in protein expression of TRPA1. (B) RT-qPCR analysis showed significant changes in TRPA1 mRNA expression. Statistical significance was denoted as *P (Bleomycin group vs. Control group, *P < 0.05, **P < 0.01, ***P < 0.001, ****P < 0.0001) and #P (Bleomycin + HC-030031 group vs. Bleomycin group, ^#^P < 0.05, ^##^P < 0.01, ^###^P < 0.001, ^####^P < 0.0001). Supplementary file1 (TIF 11941 KB)

Figure S2: Quantitative analysis by Masson staining. Masson staining of lung tissue was quantified using Halo software, n=6 per group. Statistical significance was denoted as *P (Bleomycin group vs. Control group, *P < 0.05, **P < 0.01, ***P < 0.001, ****P < 0.0001) and ^#^P (Bleomycin + HC-030031 group vs. Bleomycin group, ^#^P < 0.05, ^##^P < 0.01, ^###^P < 0.001, ^####^P < 0.0001). Supplementary file2 (TIF 31968 KB)

Figure S3: Quantitative analysis by Sirius red staining. Sirius red staining of lung tissue was quantified using Halo software, n=6 per group. Statistical significance was denoted as *P (Bleomycin group vs. Control group, *P < 0.05, **P < 0.01, ***P < 0.001, ****P < 0.0001) and #P (Bleomycin + HC-030031 group vs. Bleomycin group, ^#^P < 0.05, ^##^P < 0.01, ^###^P < 0.001, ^####^P < 0.0001). Supplementary file3 (TIF 31208 KB)

## Data Availability

Not applicable.

## Abbreviations

TRPA1: Transient receptor potential ankyrin 1
IPF: Idiopathic pulmonary fibrosis
TGF: Transforming growth factor
Smad2: Suppressor of mothers against decapentaplegic 2
CT: Computed tomography
TRP: Transient receptor potential
HE: Hematoxylin and Eosin
AFM: Atomic force microscopy
IL: Interleukin
ELISA: Enzyme-linked immunosorbent assay
CD: Cluster of differentiation
BV: Brilliant Violet™
FITC: Fluorescein isothiocyanate
PCR: Polymerase chain reaction
RT-qPCR: Real-time quantitative PCR
SDS: Sodium dodecyl-sulfate
RT: Room temperature
BSA: Bovine serum albumin
SMA: Smooth muscle actin
Ig: Immunoglobulin
H + L: Heavy chain + light chain
EEP: End-expiratory pause
EIP: End-inspiratory pause
TE: Expiration time
PENH: Enhanced pauses
MV: Minute volume
pO_2_: Partial pressure of oxygen
pCO_2_: Partial pressure of carbon dioxide
sO_2_: Oxygen saturation
ctO_2_: Total oxygen content
FO_2_Hb: Oxyhemoglobin
FHHb: Deoxyhemoglobin
EMT: Epithelial-mesenchymal transition
CaMK: Ca^2^^+^/calmodulin-dependent protein kinase
PMA: Phorbol 12-myristate 13-acetate

## References

[1] Maher TM, Bendstrup E, Dron L, Langley J, Smith G, Khalid JM (2021). Global incidence and prevalence of idiopathic pulmonary fibrosis. Respir Res.

[2] Sgalla G, Iovene B, Calvello M, Ori M, Varone F, Richeldi L (2018). Idiopathic pulmonary fibrosis: pathogenesis and management. Respir Res.

[3] Raghu G, Remy-Jardin M, Myers JL, Richeldi L, Ryerson CJ, Lederer DJ (2018). Diagnosis of idiopathic pulmonary fibrosis. An official ATS/ERS/JRS/ALAT Clinical Practice Guideline. Am J Respir Crit Care Med.

[4] Raghu G, Remy-Jardin M, Richeldi L, Thomson CC, Inoue Y, Johkoh T (2022). Idiopathic pulmonary fibrosis (an update) and progressive pulmonary fibrosis in adults: an official ATS/ERS/JRS/ALAT Clinical Practice Guideline. Am J Respir Crit Care Med.

[5] Lancaster LH, de Andrade JA, Zibrak JD, Padilla ML, Albera C, Nathan SD (2017). Pirfenidone safety and adverse event management in idiopathic pulmonary fibrosis. Eur Respir Rev.

[6] Pitre T, Mah J, Helmeczi W, Khalid MF, Cui S, Zhang M (2022). Medical treatments for idiopathic pulmonary fibrosis: a systematic review and network meta-analysis. Thorax.

[7] Ross EA, Devitt A, Johnson JR (2021). Macrophages: the good, the bad, and the gluttony. Front Immunol.

[8] Liu B, Jiang Q, Chen R, Gao S, Xia Q, Zhu J (2022). Tacrolimus ameliorates bleomycin-induced pulmonary fibrosis by inhibiting M2 macrophage polarization via JAK2/STAT3 signaling. Int Immunopharmacol.

[9] Wang J, Xu L, Xiang Z, Ren Y, Zheng X, Zhao Q (2020). Microcystin-LR ameliorates pulmonary fibrosis via modulating CD206(+) M2-like macrophage polarization. Cell Death Dis.

[10] Liu Z, Kuang W, Zhou Q, Zhang Y (2018). TGF-β1 secreted by M2 phenotype macrophages enhances the stemness and migration of glioma cells via the SMAD2/3 signalling pathway. Int J Mol Med.

[11] Wang L, Zhang Y, Zhang N, Xia J, Zhan Q, Wang C (2019). Potential role of M2 macrophage polarization in ventilator-induced lung fibrosis. Int Immunopharmacol.

[12] Bohlen CJ, Priel A, Zhou S (2010). A bivalent tarantula toxin activates the capsaicin receptor, TRPV1, by targeting the outer pore domain. Cell.

[13] Donnelly CR, Chen O, Ji RR (2020). how do sensory neurons sense danger signals?. Trends Neurosci.

[14] Marshall-Gradisnik S, Eaton-Fitch N (2022). Understanding myalgic encephalomyelitis. Science.

[15] Matsuo T, Isosaka T, Hayashi Y (2021). Thiazoline-related innate fear stimuli orchestrate hypothermia and anti-hypoxia via sensory TRPA1 activation. Nat Commun.

[16] Lin King JV, Emrick JJ, Kelly MJS (2019). A cell-penetrating scorpion toxin enables mode-specific modulation of TRPA1 and pain. Cell.

[17] Naert R, López-Requena A, Talavera K (2021). TRPA1 expression and pathophysiology in immune cells. Int J Mol Sci.

[18] Mäki-Opas I, Hämäläinen M, Moilanen E, Scotece M (2023). TRPA1 as a potential factor and drug target in scleroderma: dermal fibrosis and alternative macrophage activation are attenuated in TRPA1-deficient mice in bleomycin-induced experimental model of scleroderma. Arthritis Res Ther.

[19] Wang Z, Xu Y, Wang M, Ye J, Liu J, Jiang H (2018). TRPA1 inhibition ameliorates pressure overload-induced cardiac hypertrophy and fibrosis in mice. EBioMedicine.

[20] Okada Y, Shirai K, Reinach PS, Kitano-Izutani A, Miyajima M, Flanders KC (2014). TRPA1 is required for TGF-β signaling and its loss blocks inflammatory fibrosis in mouse corneal stroma. Lab Investig.

[21] Lou Y, Liu Y, Zhao J (2021). Activation of transient receptor potential ankyrin 1 and Vanilloid 1 channels promotes odontogenic differentiation of human dental pulp cells. J Endod.

[22] Wu Y, Duan J, Li B (2020). Exposure to formaldehyde at low temperatures aggravates allergic asthma involved in transient receptor potential ion channel. Environ Toxicol Pharmacol.

[23] Lu H, Wu L, Liu L (2018). Quercetin ameliorates kidney injury and fibrosis by modulating M1/M2 macrophage polarization. Biochem Pharmacol.

[24] Huang G, Luo J, Guo H (2022). Molybdenum and cadmium co-exposure promotes M1 macrophage polarization through oxidative stress-mediated inflammatory response and induces pulmonary fibrosis in Shaoxing ducks (*Anas platyrhyncha*). Environ Toxicol.

[25] Yao Y, Zhao X, Zheng S (2021). Subacute cadmium exposure promotes M1 macrophage polarization through oxidative stress-evoked inflammatory response and induces porcine adrenal fibrosis. Toxicology.

[26] Glass DS, Grossfeld D, Renna HA, Agarwala P, Spiegler P, DeLeon J (2022). Idiopathic pulmonary fibrosis: current and future treatment. Clin Respir J.

[27] Spagnolo P, Kropski JA, Jones MG, Lee JS, Rossi G, Karampitsakos T (2021). Idiopathic pulmonary fibrosis: disease mechanisms and drug development. Pharmacol Ther.

[28] Velagacherla V, Mehta CH, Nayak Y, Nayak UY (2022). Molecular pathways and role of epigenetics in the idiopathic pulmonary fibrosis. Life Sci.

[29] Mei Q, Liu Z, Zuo H, Yang Z, Qu J (2021). Idiopathic pulmonary fibrosis: an update on pathogenesis. Front Pharmacol.

[30] Heukels P, Moor CC, von der Thüsen JH, Wijsenbeek MS, Kool M (2019). Inflammation and immunity in IPF pathogenesis and treatment. Respir Med.

[31] Meents JE, Ciotu CI, Fischer MJM (2019). TRPA1: a molecular view. J Neurophysiol.

[32] Wang M, Zhao M, Xu S, Zheng Z, Zhang J, Pan W (2023). TRPA1 deficiency attenuates cardiac fibrosis via regulating GRK5/NFAT signaling in diabetic rats. Biochem Pharmacol.

[33] Wang M, Zhang Y, Xu M, Zhang H, Chen Y, Chung KF (2019). Roles of TRPA1 and TRPV1 in cigarette smoke -induced airway epithelial cell injury model. Free Radical Biol Med.

[34] Liu Q, Guo S, Huang Y, Wei X, Liu L, Huo F (2022). Inhibition of TRPA1 ameliorates periodontitis by reducing periodontal ligament cell oxidative stress and apoptosis via PERK/eIF2α/ATF-4/CHOP signal pathway. Oxid Med Cell Longev.

[35] Li M, Fan X, Yue Q, Hu F, Zhang Y, Zhu C (2020). The neuro-immune interaction in airway inflammation through TRPA1 expression in CD4+ T cells of asthmatic mice. Int Immunopharmacol.

[36] Dietrich A (2019). Modulators of transient receptor potential (TRP) Channels as therapeutic options in lung disease. Pharmaceuticals (Basel, Switzerland).

[37] Wang Z, Wang M, Liu J, Ye J, Jiang H, Xu Y (2018). Inhibition of TRPA1 attenuates doxorubicin-induced acute cardiotoxicity by suppressing oxidative stress, the inflammatory response, and endoplasmic reticulum stress. Oxid Med Cell Longev.

[38] Kuznetsova TG, Starodubtseva MN, Yegorenkov NI, Chizhik SA, Zhdanov RI (2007). Atomic force microscopy probing of cell elasticity. Micron (Oxford, England 1993).

[39] Guz N, Dokukin M, Kalaparthi V, Sokolov I (2014). If cell mechanics can be described by elastic modulus: study of different models and probes used in indentation experiments. Biophys J.

[40] Reghelin CK, Bastos MS, de Souza BB, Costa BP, Lima KG, de Sousa AC (2023). Bezafibrate reduces the damage, activation and mechanical properties of lung fibroblast cells induced by hydrogen peroxide. Naunyn Schmiedebergs Arch Pharmacol.

[41] Zhang L, Wang Y, Wu G, Xiong W, Gu W, Wang CY (2018). Macrophages: friend or foe in idiopathic pulmonary fibrosis?. Respir Res.

[42] Chang X, Xing L, Wang Y, Zhou TJ, Shen LJ, Jiang HL (2020). Nanoengineered immunosuppressive therapeutics modulating M1/M2 macrophages into the balanced status for enhanced idiopathic pulmonary fibrosis therapy. Nanoscale.

[43] Kishore A, Petrek M (2021). Roles of macrophage polarization and macrophage-derived mirnas in pulmonary fibrosis. Front Immunol.

[44] Hou J, Shi J, Chen L, Lv Z, Chen X, Cao H (2018). M2 macrophages promote myofibroblast differentiation of LR-MSCs and are associated with pulmonary fibrogenesis. Cell Commun Signal.

[45] Wang Q, Chen K, Zhang F, Peng K, Wang Z, Yang D (2020). TRPA1 regulates macrophages phenotype plasticity and atherosclerosis progression. Atherosclerosis.

[46] Wynn TA, Barron L (2010). Macrophages: master regulators of inflammation and fibrosis. Semin Liver Dis.

[47] Nakamura R, Bing R, Gartling GJ, Branski RC (2022). Macrophages alter inflammatory and fibrotic gene expression in human vocal fold fibroblasts. Exp Cell Res.

[48] Sharma L, Wu W, Dholakiya SL, Gorasiya S, Wu J, Sitapara R (2014). Assessment of phagocytic activity of cultured macrophages using fluorescence microscopy and flow cytometry. Methods Mol Biol (Clifton, NJ).

[49] Rao LZ, Wang Y, Zhang L, Wu G, Zhang L, Wang FX (2021). IL-24 deficiency protects mice against bleomycin-induced pulmonary fibrosis by repressing IL-4-induced M2 program in macrophages. Cell Death Differ.

[50] Ning J, Ye Y, Bu D, Zhao G, Song T, Liu P (2021). Imbalance of TGF-β1/BMP-7 pathways induced by M2-polarized macrophages promotes hepatocellular carcinoma aggressiveness. Mol Ther.

[51] Su N, Xiao C, Wei Y, Kou Q, Jiang Z (2018). The role of ERK and Smad2 signal pathways in the alternatively activated macrophages induced by TGF-β1 and high-ambient glucose. J Recept Signal Transduct Res.

[52] Chen B, Li R, Hernandez SC, Hanna A, Su K, Shinde AV (2022). Differential effects of Smad2 and Smad3 in regulation of macrophage phenotype and function in the infarcted myocardium. J Mol Cell Cardiol.

[53] Ying H, Fang M, Hang QQ, Chen Y, Qian X, Chen M (2021). Pirfenidone modulates macrophage polarization and ameliorates radiation-induced lung fibrosis by inhibiting the TGF-β1/Smad3 pathway. J Cell Mol Med.

[54] Zhao M, Ding N, Wang H, Zu S, Liu H, Wen J (2023). Activation of TRPA1 in bladder suburothelial myofibroblasts counteracts TGF-β1-induced fibrotic changes. Int J Mol Sci.

[55] Yap JMG, Ueda T, Takeda N, Fukumitsu K, Fukuda S, Uemura T (2020). An inflammatory stimulus sensitizes TRPA1 channel to increase cytokine release in human lung fibroblasts. Cytokine.

[56] Virk HS, Biddle MS, Smallwood DT, Weston CA, Castells E, Bowman VW (2021). TGFβ1 induces resistance of human lung myofibroblasts to cell death via down-regulation of TRPA1 channels. Br J Pharmacol.

[57] Geiger F, Zeitlmayr S, Staab-Weijnitz CA, Rajan S, Breit A, Gudermann T (2023). An inhibitory function of TRPA1 channels in TGF-β1-driven fibroblast-to-myofibroblast differentiation. Am J Respir Cell Mol Biol.

[58] Scheraga RG, Olman MA (2023). TRP channels in pulmonary fibrosis: variety is a spice of life. Am J Respir Cell Mol Biol.

