# Supplementary figures and images for: Role of transient receptor potential ankyrin 1 in idiopathic pulmonary fibrosis: modulation of M2 macrophage polarization
Source: Cell Mol Life Sci. 2024 Apr 18;81(1):187. doi: 10.1007/s00018-024-05219-x (PMC11026287; doi:10.1007/s00018-024-05219-x)

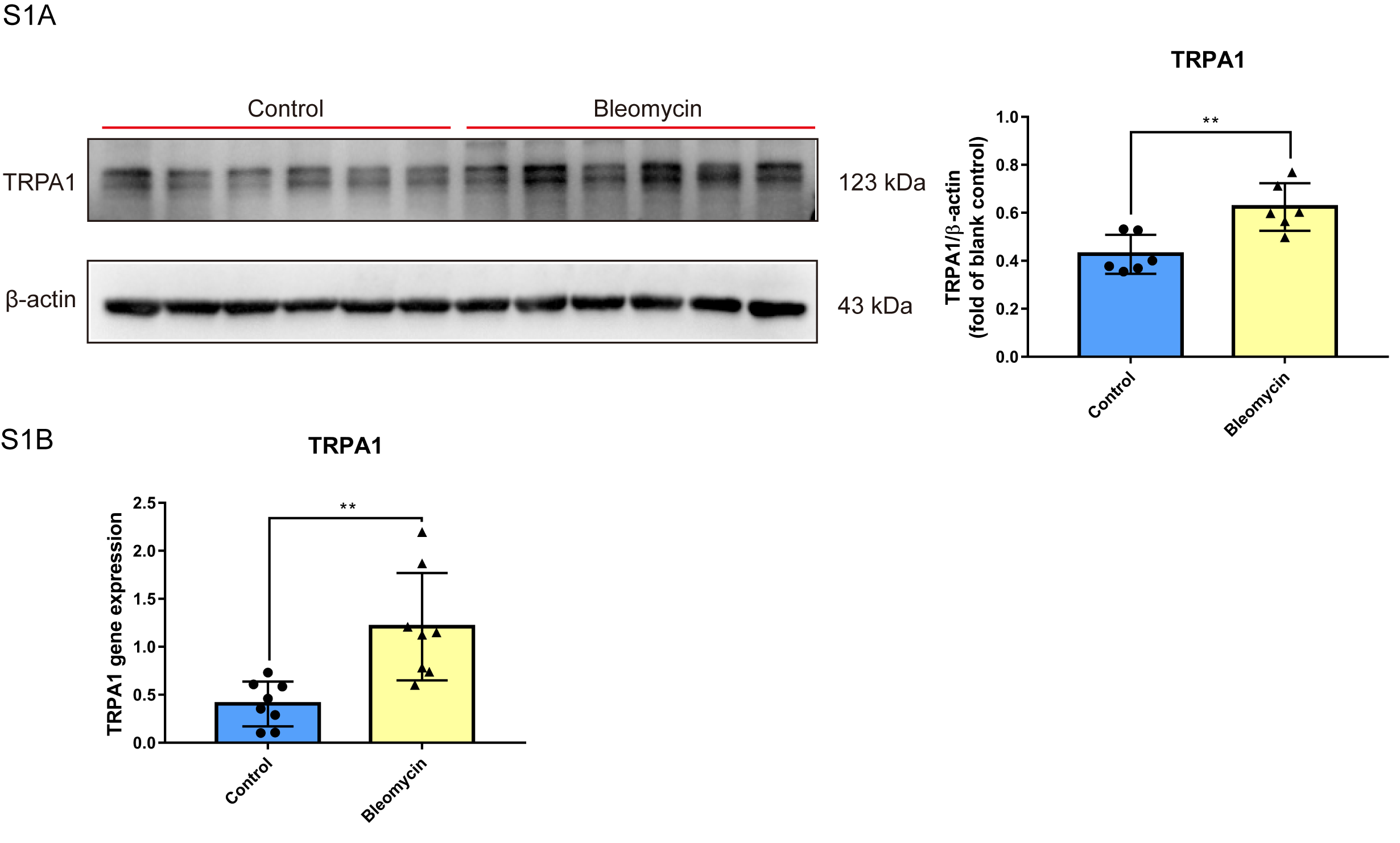

Supplement: Supplementary file 1 — Figure S1: TRPA1 expression is increased in mice of bleomycin-induced pulmonary fibrosis. (A) Western blot analysis showed significant changes in protein expression of TRPA1. (B) RT-qPCR analysis showed significant changes in TRPA1 mRNA expression. Statistical significance was denoted as *P (Bleomycin group vs. Control group, *P < 0.05, **P < 0.01, ***P < 0.001, ****P < 0.0001) and #P (Bleomycin + HC-030031 group vs. Bleomycin group, #P < 0.05, ##P < 0.01, ###P < 0.001, ####P < 0.0001). Supplementary file1 (TIF 11941 KB) [file 18_2024_5219_MOESM1_ESM.tif]

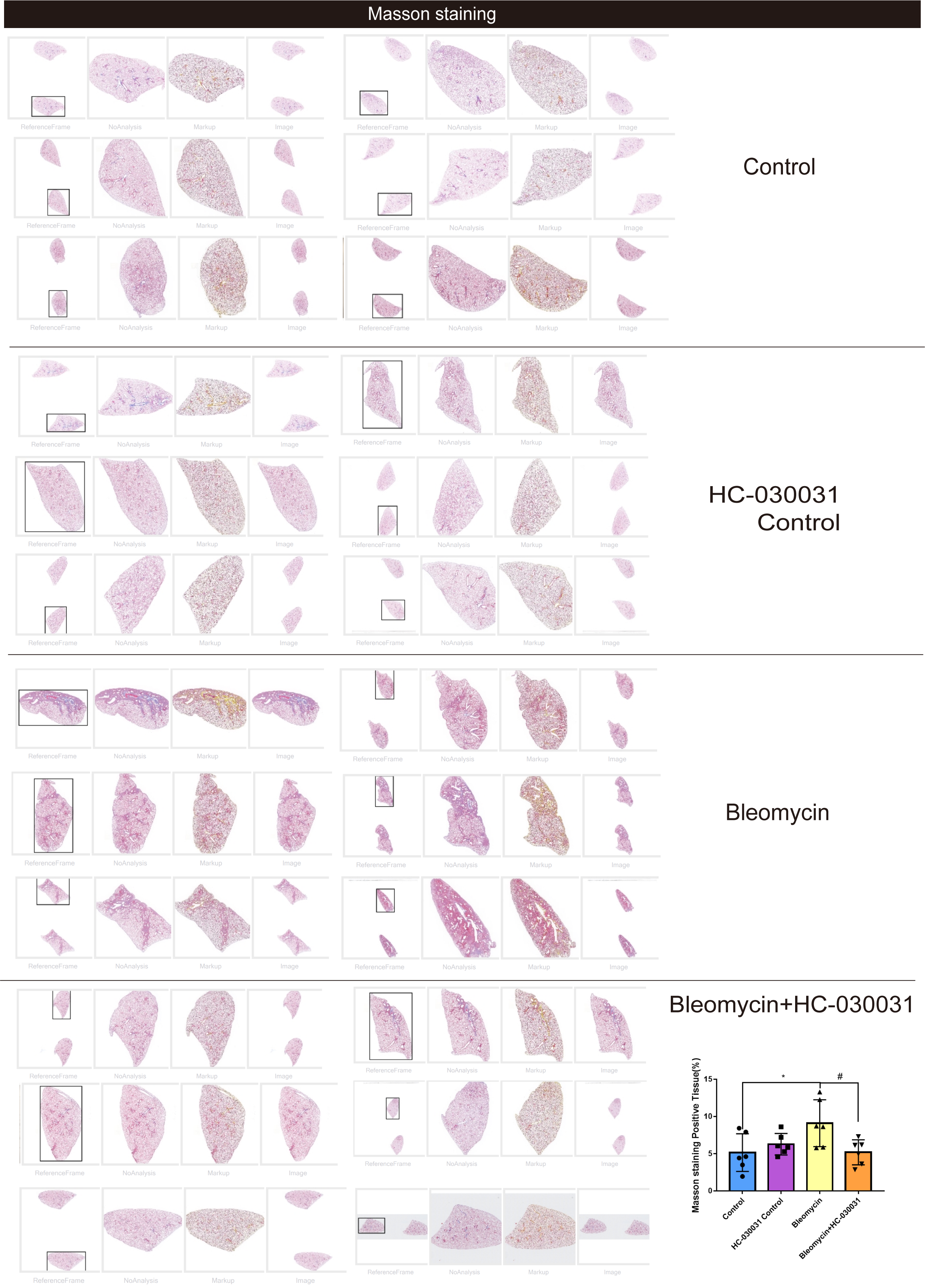

Supplement: Supplementary file 2 — Figure S2: Quantitative analysis by Masson staining. Masson staining of lung tissue was quantified using Halo software, n=6 per group. Statistical significance was denoted as *P (Bleomycin group vs. Control group, *P < 0.05, **P < 0.01, ***P < 0.001, ****P < 0.0001) and #P (Bleomycin + HC-030031 group vs. Bleomycin group, #P < 0.05, ##P < 0.01, ###P < 0.001, ####P < 0.0001). Supplementary file2 (TIF 31968 KB) [file 18_2024_5219_MOESM2_ESM.tif]

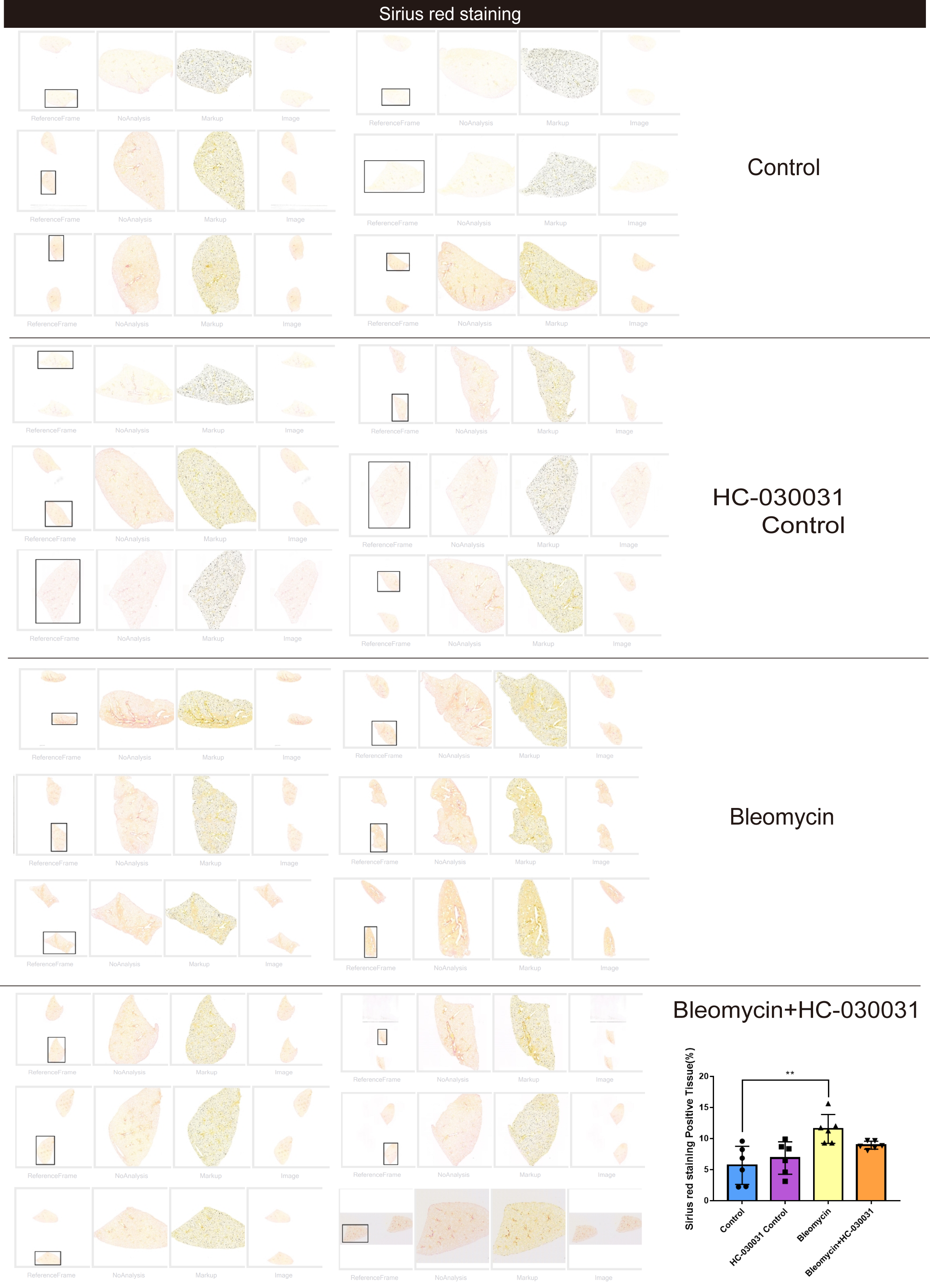

Supplement: Supplementary file 3 — Figure S3: Quantitative analysis by Sirius red staining. Sirius red staining of lung tissue was quantified using Halo software, n=6 per group. Statistical significance was denoted as *P (Bleomycin group vs. Control group, *P < 0.05, **P < 0.01, ***P < 0.001, ****P < 0.0001) and #P (Bleomycin + HC-030031 group vs. Bleomycin group, #P < 0.05, ##P < 0.01, ###P < 0.001, ####P < 0.0001). Supplementary file3 (TIF 31208 KB) [file 18_2024_5219_MOESM3_ESM.tif]
